# Heme sensing and detoxification by HatRT contributes to pathogenesis during *Clostridium difficile* infection

**DOI:** 10.1371/journal.ppat.1007486

**Published:** 2018-12-21

**Authors:** Reece J. Knippel, Joseph P. Zackular, Jessica L. Moore, Arianna I. Celis, Andy Weiss, M. Kay Washington, Jennifer L. DuBois, Richard M. Caprioli, Eric P. Skaar

**Affiliations:** 1 Department of Pathology, Microbiology and Immunology, Vanderbilt University Medical Center, Nashville, TN, United States of America; 2 Vanderbilt Institute for Infection, Immunology and Inflammation, Vanderbilt University Medical Center, Nashville, TN, United States of America; 3 Department of Biochemistry, Vanderbilt University, Nashville, TN, United States of America; 4 Department of Chemistry and Biochemistry, Montana State University, Bozeman, MT, United States of America; 5 Department of Chemistry, Vanderbilt University, Nashville, TN, United States of America; University of Texas Medical School at Houston, UNITED STATES

## Abstract

*Clostridium difficile* is a Gram-positive, spore-forming anaerobic bacterium that infects the colon, causing symptoms ranging from infectious diarrhea to fulminant colitis. In the last decade, the number of *C*. *difficile* infections has dramatically risen, making it the leading cause of reported hospital acquired infection in the United States. Bacterial toxins produced during *C*. *difficile* infection (CDI) damage host epithelial cells, releasing erythrocytes and heme into the gastrointestinal lumen. The reactive nature of heme can lead to toxicity through membrane disruption, membrane protein and lipid oxidation, and DNA damage. Here we demonstrate that *C*. *difficile* detoxifies excess heme to achieve full virulence within the gastrointestinal lumen during infection, and that this detoxification occurs through the heme-responsive expression of the heme activated transporter system (HatRT). Heme-dependent transcriptional activation of *hatRT* was discovered through an RNA-sequencing analysis of *C*. *difficile* grown in the presence of a sub-toxic concentration of heme. HatRT is comprised of a TetR family transcriptional regulator (*hatR)* and a major facilitator superfamily transporter (*hatT*). Strains inactivated for *hatR* or *hatT* are more sensitive to heme toxicity than wild-type. HatR binds heme, which relieves the repression of the *hatRT* operon, whereas HatT functions as a heme efflux pump. In a murine model of CDI, a strain inactivated for *hatT* displayed lower pathogenicity in a toxin-independent manner. Taken together, these data suggest that HatR senses intracellular heme concentrations leading to increased expression of the *hatRT* operon and subsequent heme efflux by HatT during infection. These results describe a mechanism employed by *C*. *difficile* to relieve heme toxicity within the host, and set the stage for the development of therapeutic interventions to target this bacterial-specific system.

## Author summary

*Clostridium difficile* is a pathogenic bacterium that infects the colon and is the leading cause of infectious diarrhea in the United States. *C*. *difficile* mediated disease is driven by the production of two toxins, TcdA and TcdB. The toxins cause severe damage to the intestinal epithelial layer of the colon resulting in inflammation and bleeding. Once in the intestinal lumen, red blood cells lyse, resulting in an abundance of extracellular heme at the site of *C*. *difficile* infection. Due to the highly reactive nature of heme, elevated concentrations are toxic to bacteria. Here we describe a *C*. *difficile* system that allows for survival in the presence of heme intoxication. These results present a mechanism employed by *C*. *difficile* to sense and reduce intracellular heme concentrations to relieve toxicity. We also demonstrate the importance of heme detoxification during *C*. *difficile* infection, as strains lacking this system display reduced virulence in a murine model of infection. Collectively, these data provide insight into a mechanism that *C*. *difficile* utilizes to survive in the host environment during infection.

## Introduction

*Clostridium difficile* is a spore-forming, Gram-positive obligate anaerobe that is the most common cause of nosocomial infections in the United States [1]. *C*. *difficile* infects the colon, causing a wide range of diseases that vary from infectious diarrhea to pseudomembranous colitis. During infection, *C*. *difficile* produces two potent toxins, TcdA and TcdB, which cause severe damage to intestinal epithelial cells resulting in inflammation, fluid secretion, and necrotic cell death [2, 3].

Perforations in the intestinal epithelial layer lead to bleeding in the gut and subsequent translocation of erythrocytes into the gastrointestinal lumen [4]. Hemolysis due to pathophysiological stress occurs, resulting in the release of hemoglobin-bound heme and free heme at the site of damage [5]. Heme, an iron-containing porphyrin, is the most abundant source of iron in the human body and many invading pathogens have evolved mechanisms to utilize this rich metabolic resource [6–10]. Owing to its reactive nature, heme is toxic to bacteria at high concentrations through a variety of mechanisms [11–14]. In order to defend against the stresses of heme-mediated damage, bacteria encode systems for heme sensing and detoxification [15–21]. In the Gram-positive pathogens *Staphylococcus aureus* and *Bacillus anthracis*, the heme stress response is controlled by the heme sensing two-component system, HssRS, which regulates transcription of the ABC transporter HrtAB to reduce heme toxicity through efflux [15, 16, 22]. Reducing intracellular heme levels through export is a conserved microbial strategy as heme efflux systems have also been identified in *Lactococcus lactis*, *Streptococcus agalactiae*, and *Neisseria gonorrhoeae* [17, 18, 23]. In each example, inactivation of heme detoxification machinery increases heme sensitivity and modulates virulence [15–18, 23]. Notably, *C*. *difficile* does not contain orthologs of known heme detoxification systems, and it is also unknown if this organism encounters heme during infection.

The overall goal of this study was to investigate the occurrence of heme exposure to *C*. *difficile* within the gastrointestinal lumen during infection. Here, we visualize increased abundance of hemoglobin in the gastrointestinal lumen as a result of CDI using imaging mass spectrometry. A heme-inducible operon was identified that contains a TetR family transcriptional regulator and major-facilitator superfamily transporter. We have named these gene products HatRT for heme activated transporter (R = regulator, T = transporter). The transcriptional regulator HatR responds to intracellular heme concentrations through binding of heme leading to the de-repression and increased transcription of *hatRT*. Lack of the HatT transporter results in increased intracellular heme concentrations and a decrease in pathogenicity in a murine model of infection. Taken together, these results describe a mechanism by which *C*. *difficile* detoxifies heme and establishes a requirement for heme sensing and detoxification for full virulence during *C*. *difficile* infection.

## Results

### *C*. *difficile* infection increases hemoglobin abundance in the gastrointestinal lumen

**Fig 1:**
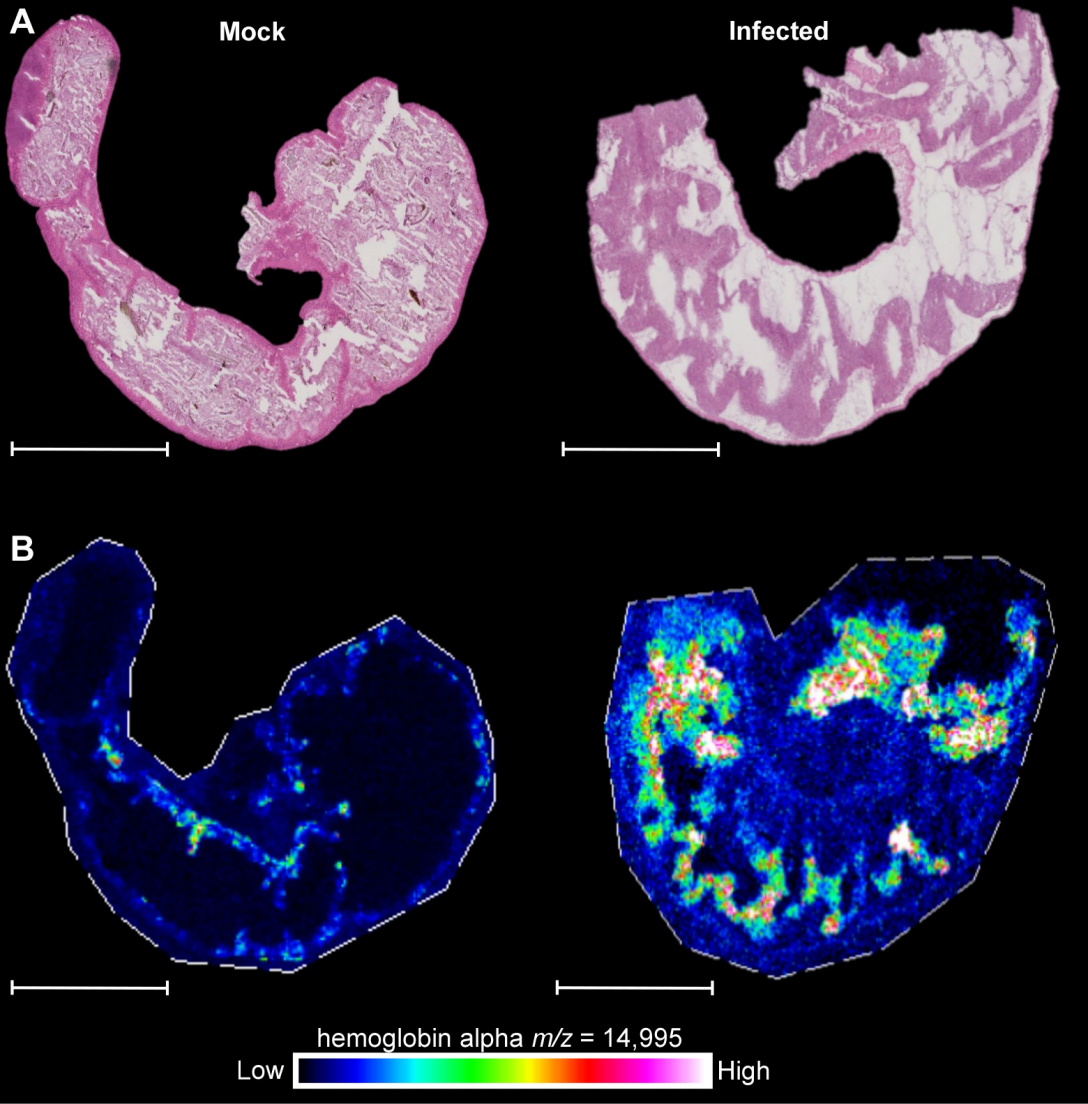
Hemoglobin accumulates in the cecum during *C*. *difficile* infection. (A) Representative H&E images of mock-infected and *C*. *difficile* strain R20291 infected C57BL/6 mice. (B) Abundance of hemoglobin subunit alpha in serial sections of the same ceca determine by MALDI IMS. Each image is a representative of 5 independent ceca. Scale bars, 5 mm.

To identify host proteins that increase in abundance during *C*. *difficile* infection (CDI), we applied matrix-assisted laser desorption ionization imaging mass spectrometry (MALDI IMS) to a murine model that induces susceptibility to infection through administration of cefoperazone (0.5 mg/mL) [24–27]. Ceca of mice infected with *C*. *difficile* R20291 presented with high levels of epithelial damage, edema, and inflammation on day 4 of the infection (Fig 1A). This inflammatory response correlated with a high abundance of the alpha chain of hemoglobin at the sites of pathology and in the luminal space (Fig 1B and S1 Fig). In contrast, mouse ceca mock infected with PBS did not exhibit pathology (Fig 1A) and displayed a low abundance of hemoglobin alpha concentrated at the periphery of the intestinal epithelial villi (Fig 1B and S1 Fig). These data demonstrate that CDI leads to high concentrations of hemoglobin at this host-pathogen interface, and considering each hemoglobin protein contains four molecules of heme, support a model whereby *C*. *difficile* experiences heme stress during infection.

### The transcriptional response of *C*. *difficile* to heme exposure

**Fig 2:**
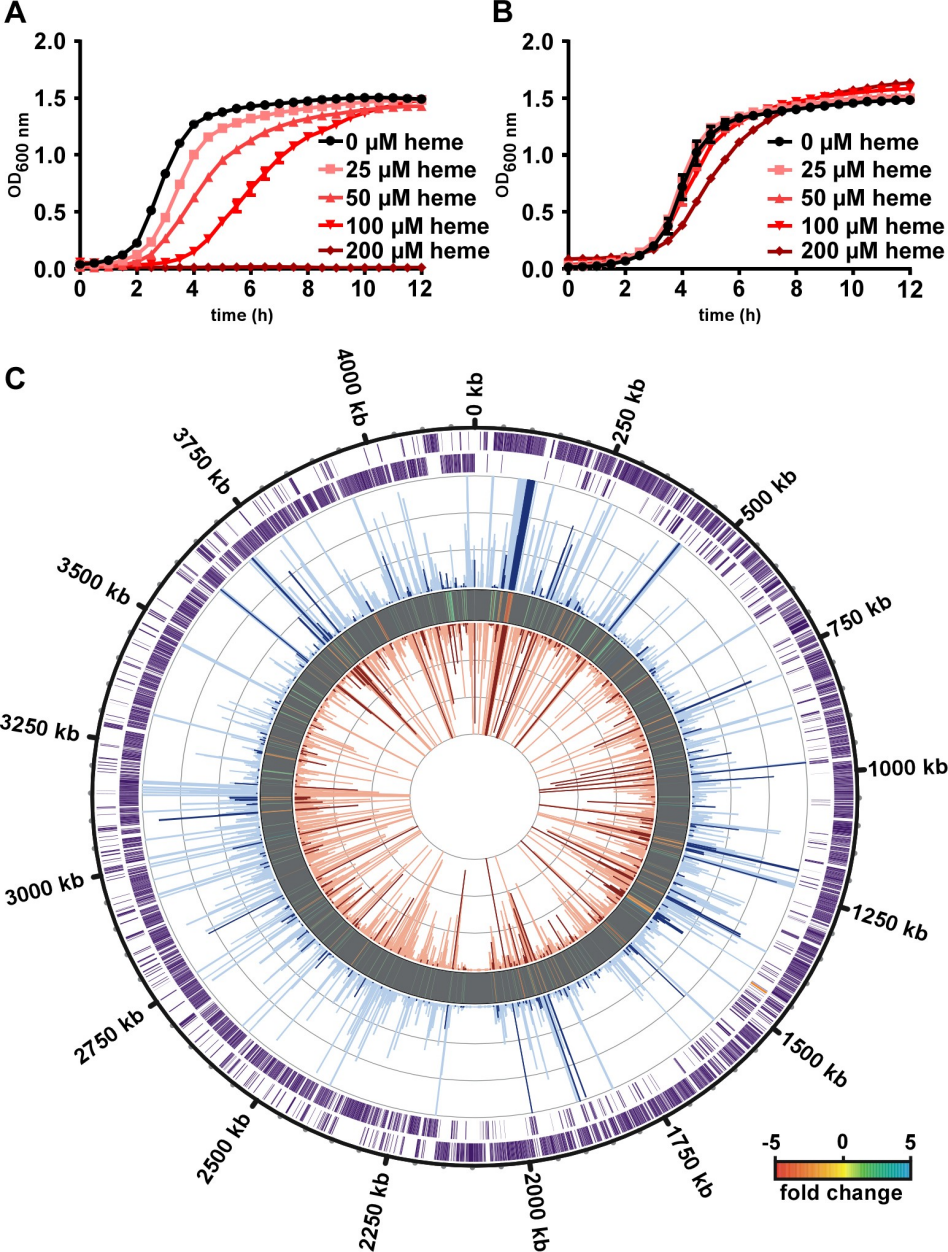
*C*. *difficile* encodes machinery to detoxify heme. (A) Growth of *C*. *difficile* R20291 in CDMM containing increasing concentrations of heme. (B) Growth of overnight heme treated (1 μM) *C*. *difficile* in CDMM containing the exact concentrations of heme as in A. For A and B, the data are the average of means from at least three independent experiments each in biological triplicate with standard error of the mean shown. (C) RNA-sequencing analysis comparing RNA from heme treated (50 μM, total transcript abundance shown in red peaks inside circle where dark red peaks are significantly changed genes) *C*. *difficile* to an untreated control (total transcript abundance shown in blue peaks middle circle where dark blue peaks are significantly changed genes). Fold change differences are shown in the circle between the control and heme treated samples with a 5-fold cut off depicted according to the indicated heap map. Outside two rims represent genes in the *C*. *difficile* R20291 genome (outside is coded in the forward direction, inside is coded in the reverse direction, orange denotes *hatRT* operon).

Considering the high concentration of hemoglobin in the infected lumen and the reactive nature of heme, we investigated the sensitivity of *C*. *difficile* to heme toxicity [13]. When *C*. *difficile* was grown over time in increasing concentrations of heme (0–200 μM), a dose-dependent increase in toxicity was observed with a complete inhibition of growth at the highest concentration (Fig 2A). To determine if *C*. *difficile* can adapt to heme exposure, the growth of *C*. *difficile* cells pre-exposed to a low concentration of heme (1 μM) was measured following sub-culturing into media containing varying concentrations of heme (0–200 μM; Fig 2B). Heme pre-exposure corrected the growth defects of *C*. *difficile* cultures not pre-exposed to heme (Fig 2A and 2B), suggesting that *C*. *difficile* has an inducible mechanism for heme detoxification.

**Fig 3:**
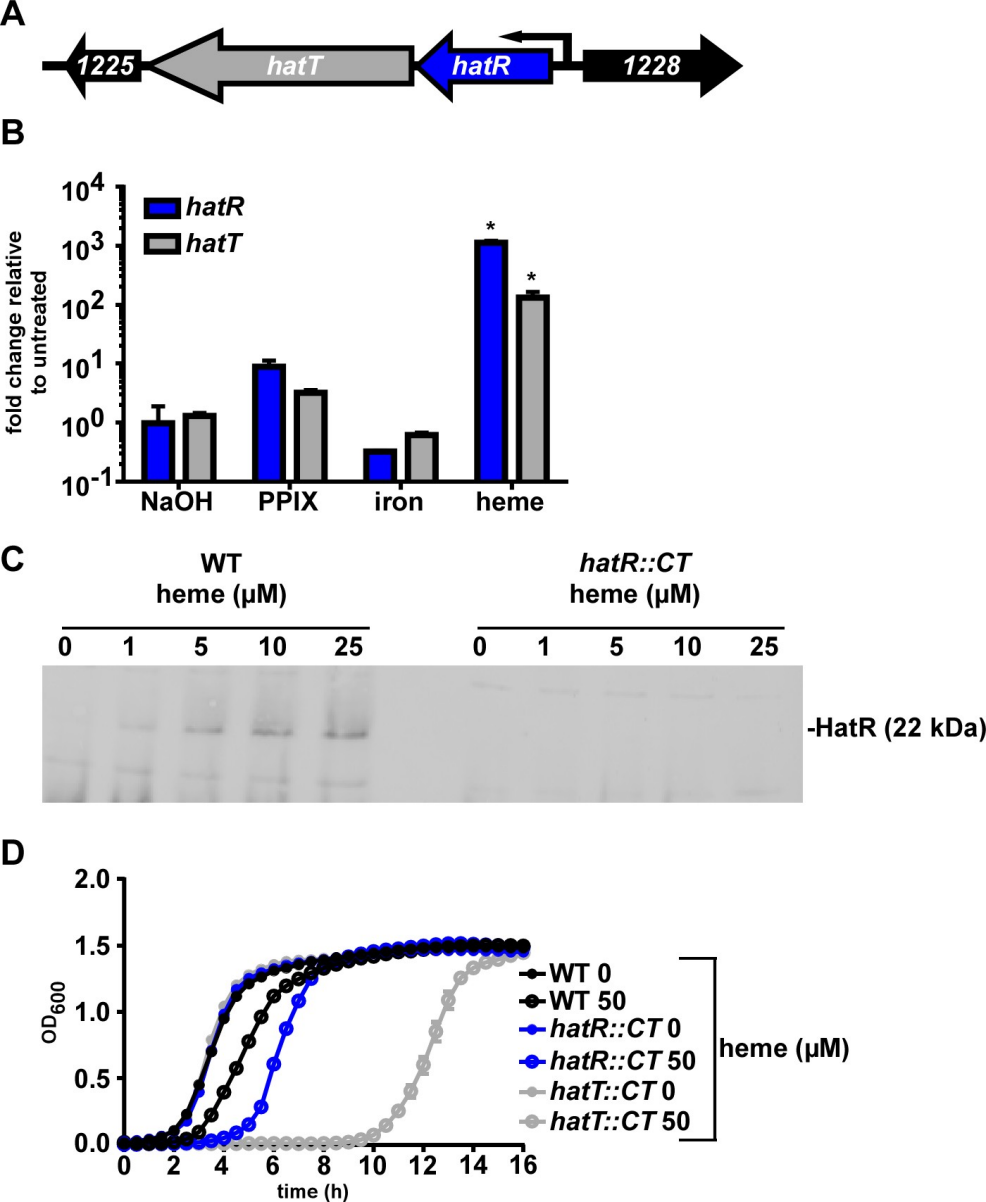
The *hatRT* operon responds to and relieves heme toxicity. (A) Schematic of the *hatRT* operon. (B) *hatR* and *hatT* transcription determined by qRT-PCR. cDNA was reverse transcribed from RNA harvested from *C*. *difficile* R20291 grown in the presence of sodium hydroxide (NaOH, 500 μM), protoporphyrin IX (PPIX, 50 μM), iron sulfate (50 μM) or heme (50 μM). Transcription is graphed as the fold change relative to an untreated control. The data are a representative of three independent experiments each in biological triplicate with standard deviation shown. Statistical significance was determined using the multiple comparison one-way ANOVA test comparing the means of each group to one another * denotes *p* < 0.001 (C) Immunoblot for HatR from *C*. *difficile* WT and *hatR*::*CT* whole cell lysates grown in the presence of increasing concentrations of heme (0–25 μM). Blots are representative of three independent experiments. (D) Growth of WT, *hatR*::*CT*, and *hatT*::*CT* strains in the presence or absence of heme (50 μM). The data are a representative from three independent experiments each in biological triplicate with standard error of the mean.

In order to identify the genes that encode proteins responsible for heme adaption, we performed an RNA-sequencing experiment comparing the total relative mRNA transcript abundance of early exponential phase (OD_600_ = 0.3) untreated cultures of *C*. *difficile* to cultures grown in 50 μM heme. Heme induced the transcription of 245 genes and decreased the transcription of 146 genes (Fig 2C; S3 and S4 Tables). This dataset was curated by grouping significantly upregulated genes that could function as a mechanism of heme sensing and detoxification. Within this group an operon of two genes encoding a TetR family transcriptional regulator (CDR20291_1227) and a major facilitator super family (MFS) transporter (CDR20291_1226) were identified as candidates for further investigation (Fig 3A). These results demonstrate that *C*. *difficile* has heme responsive genes that may account for its ability to resist and adapt to heme toxicity.

### The *hatRT* operon increases expression in response to heme and confers heme resistance

To confirm that CDR20291_1227 and CDR20291_1226 are heme responsive, cultures were grown in equimolar concentrations of NaOH (vehicle), protoporphyrin IX (porphyrin ring without iron), iron sulfate, or heme prior to harvesting RNA at the early exponential phase of growth (OD_600_ = 0.3). Quantitative reverse transcription PCR (qRT-PCR) was performed on cDNA generated from these samples using primers specific for the genes within this operon. Transcription of both genes were minimally increased in the samples treated with NaOH, protoporphyrin IX, and iron sulfate in contrast to a 2–3 log increase in transcript abundance of the heme treated samples compared to the untreated control (Fig 3B). Due to this considerable transcriptional response to heme, as well as data described below, we named CDR20291_1227 heme activated transporter regulator (*hatR*) and CDR20291_1226 heme activated transporter (*hatT*).

To investigate the heme responsive abundance of HatR, polyclonal antiserum was generated against recombinant HatR and immunoblot analyses were performed on whole cell lysates grown in increasing concentrations of heme. The increase in HatR protein abundance correlated with the increase in concentration of heme, further supporting the observation that *hatRT* is up-regulated upon heme exposure (Fig 3C). To demonstrate the specificity of this antisera, we generated a strain of *C*. *difficile* inactivated for *hatR* (*hatR*::*CT*) using the ClosTron system [28]. In this strain, HatR is no longer produced in response to heme (Fig 3C). The lack of *hatR* renders the bacteria more sensitive to heme toxicity, as growth over time in the presence of 50 μM heme is delayed in the mutant compared to wild-type (WT; Fig 3D). A more significant growth delay is observed when *hatT* is inactivated (*hatT*::*CT*) using the ClosTron system and exposed to the same concentration of heme (Fig 3D). The growth of *hatR*::*CT* and *hatT*::*CT* strains are restored to WT levels by expressing *hatR* or *hatT*, respectively, *in trans* under the control of the intergenic region upstream of *hatR* (S2 Fig). Together these data suggest that HatR and HatT coordinate to sense, respond to, and alleviate heme toxicity.

### HatR functions as a transcriptional repressor of the *hatRT* operon

**Fig 4:**
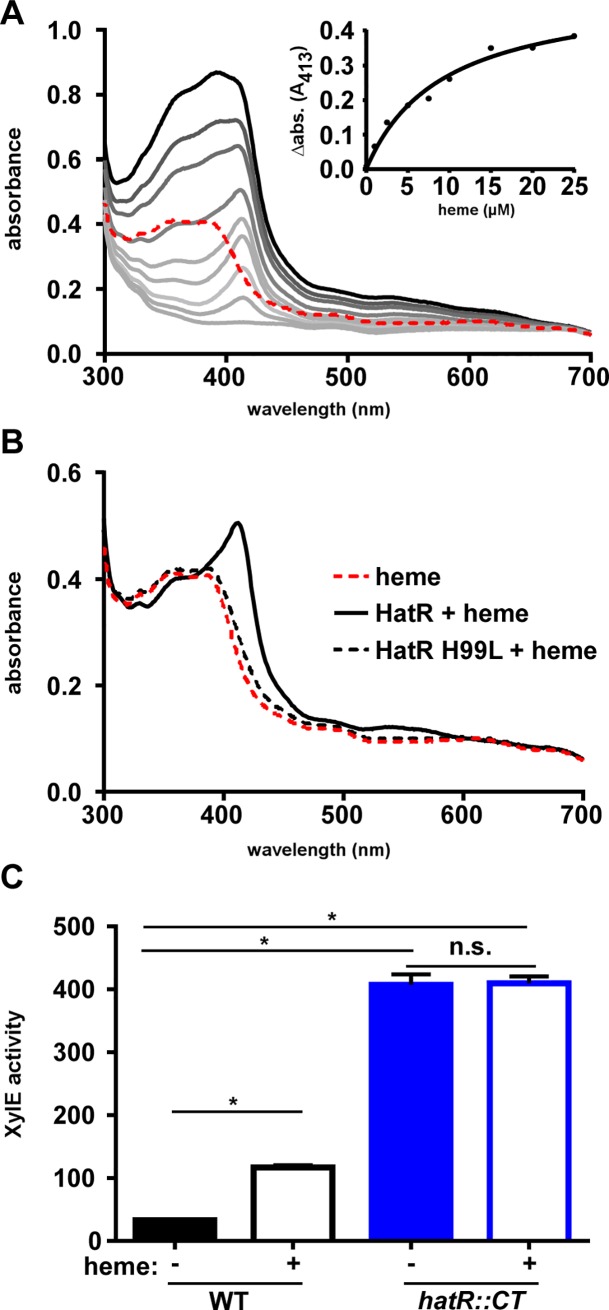
HatR transcriptional repression of the *hatRT* operon is released through direct heme binding. (A) Absorption spectra of heme binding to recombinant HatR. Increasing concentrations of heme (2.5 to 25 μM) were added to 10 μM protein. The spectrum corresponding to 25 μM heme is shown as a dashed red line. HatR with increasing concentrations of heme are shown as gray lines. The inset displays change in absorbance at 413 nm for HatR bound to heme minus the corresponding heme alone peak. (B) Absorption spectra of 10 μM heme binding to HatR and HatR H99L. (C) XylE catechol oxidase activity was measured in *C*. *difficile* WT and *hatR*::*CT* strains harboring a *hatR* promoter XylE reporter plasmid after growth in vehicle or 10 μM heme. The data are an average from three independent experiments each in biological triplicate with standard deviation. Statistical significance was determined using the multiple comparison one-way ANOVA test with the Tukey correction for multiple comparisons comparing the means of each group to one another * denotes *p <* 0.001, n.s. denotes not significant.

As most members of the TetR family of transcriptional regulators directly bind their effector molecules, we examined the ability of HatR to bind heme [29]. Recombinant HatR (10 μM) was incubated with heme (0–25 μM), resulting in the appearance of a Soret peak at 413 nm (Fig 4A), indicative of HatR-heme complex formation [30]. Differential absorption spectroscopy at 413 nm over a range of heme concentrations was used to determine that HatR binds heme at a 1:1 ratio using a single site binding model (k_d_ = 9.2 ± 1.8 μM; Fig 4A insert). To identify the residues responsible for heme binding by HatR, each histidine within HatR was individually mutated. Histidine residues were chosen for substitution due to histidines commonly serving as axial ligands that bind heme [17]. Recombinant proteins containing each individual histidine substitution were purified and heme binding was measured. Substitution of histidine 99 to leucine (H99L) was sufficient to abrogate heme binding (Fig 4B). The substitutions of the remaining four histidines to alanine or leucine (H121A, H126L, H165A, and H180A; S3 Fig) did not significantly alter heme binding. These data specify histidine 99 as a critical residue in the formation of the HatR-heme complex.

An examination into the regulation of the *hatRT* operon was performed by creating a plasmid containing a fusion of the intergenic region prior to *hatR* to the reporter gene *xylE*, and transforming this plasmid into WT *C*. *difficile* [22]. Exposure of this reporter strain to 10 μM heme led to a significant increase in XylE activity as compared to an untreated control (Fig 4C), indicating that heme treatment induces the transcription of the *hatRT* operon in the WT strain. However, upon transformation of the reporter plasmid into the *hatR*::*CT* strain, there was no significant difference in XylE activity between the untreated or heme exposed samples (Fig 4C). Moreover, the level of XylE activity of the untreated *hatR*::*CT* strain was significantly higher than the heme-exposed WT strain, suggesting constitutive expression of *xylE* in the absence of HatR. Taken together, these data suggest that HatR functions as a transcriptional repressor of the *hatRT* operon and that de-repression is achieved through the formation of a HatR-heme complex.

### HatT reduces intracellular heme concentrations

**Fig 5:**
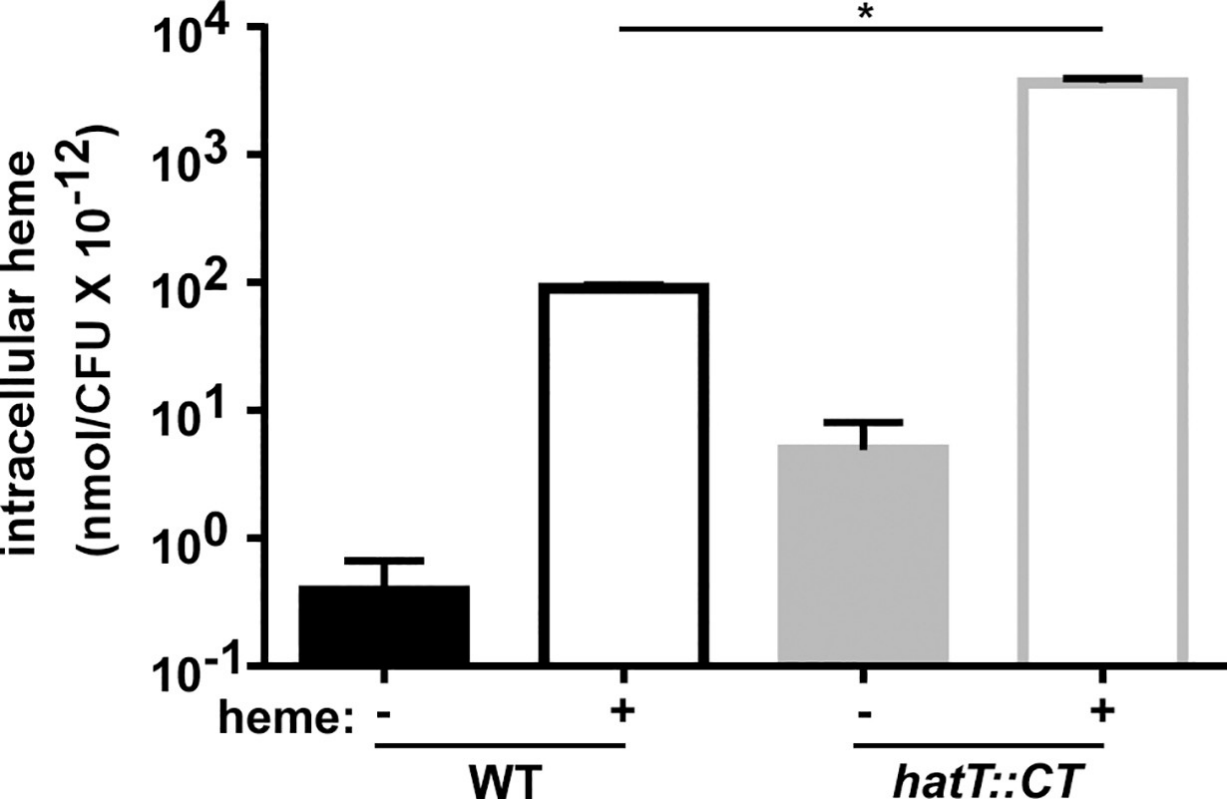
HatT reduces intracellular heme concentrations. WT and *hatT*::*CT* strains were grown to saturation in either BHIS or BHIS supplemented with 25 μM heme and harvested. Cells were analyzed for their heme content by high resolution MS. Peak areas from extracted-ion chromatograms of heme that accrued above baseline were compared to a standard curve and used to obtain nmoles per CFU. The data are the average of a single experiment performed in biological triplicate with standard deviation. Statistical significance was determined using a multiple comparison one-way ANOVA test with the Tukey correction for multiple comparisons comparing the means of each group to one another. * denotes *p* < 0.0001.

One strategy for microbial heme detoxification involves the reduction of intracellular heme concentrations through efflux [15–18, 23]. To investigate if heme efflux is responsible for HatT-dependent resistance to heme toxicity, we grew the WT and *hatT*::*CT* strains in the presence or absence of heme (25 μM) for 16 h and measured intracellular heme concentrations utilizing LC-MS analysis. The WT strain treated with heme exhibited a two-log increase in intracellular heme levels when compared to untreated WT cells (Fig 5). In contrast, a more dramatic trend was observed in the *hatT*::*CT* strain, which exhibited a three-log increase in intracellular heme concentration when compared to the *hatT*::*CT* untreated culture (Fig 5). The intracellular heme concentration was over 40-fold higher in the *hatT*::*CT* strain treated with heme when compared to the WT strain treated with heme (Fig 5). These data, combined with the heme sensitivity of the *hatT*::*CT* strain, suggest that the function of HatT is to reduce intracellular heme concentrations to relieve heme toxicity.

### HatT promotes pathogenicity in a mouse model of CDI

**Fig 6:**
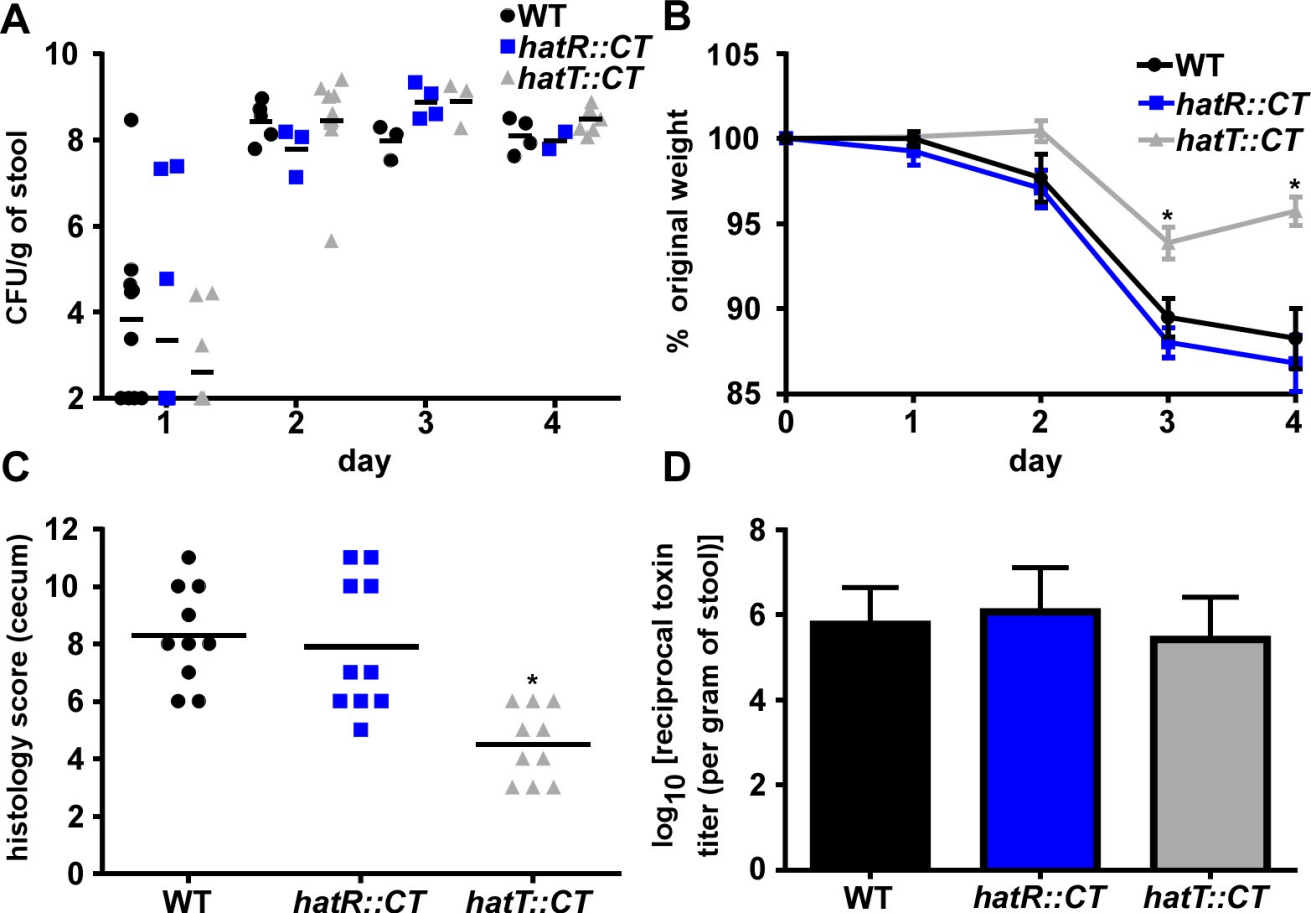
*hatT*::*CT* displays reduced pathogenicity in a mouse model of CDI. CFU analysis (A) and weights (B) of mice infected with *C*. *difficile* R20291 WT, *hatR*::*CT*, and *hatT*::*CT* strains with standard error on the mean (n = 10/group). (C) Blinded histology scoring of ceca and (D) *C*. *difficile* toxin titer per gram of feces was measured on day 4 of the infection. All of the data are represented as median or mean with standard error of the mean. Statistical significance was determined using the multiple comparison Kruskal-Wallis test with the Dunn’s correction for multiple comparisons comparing the means of each group to one another. * denotes *p* < 0.05.

The abundance of heme in the lumen during infection combined with the observed functions of HatR and HatT to sense and reduce heme concentrations, suggest that strains lacking these proteins may have reduced pathogenicity during CDI. To test this, mice were infected with WT, *hatR*::*CT*, or *hatT*::*CT* spores and disease was monitored for 4 days. All strains were able to fully colonize the mice as exhibited by ~10^8^ colony-forming units (CFU) per gram of stool (Fig 6A). Mice infected with the *hatT*::*CT* strain lost significantly less weight than the mice infected with the WT or *hatR*::*CT* strains on days 3 and 4 of the infection (Fig 6B), indicating that the mice infected with the *hatT*::*CT* strain were partially protected despite similar colonization levels. Furthermore, cecal pathology was significantly reduced in mice infected with the *hatT*::*CT* strain compared to mice infected with WT or *hatR*::*CT* strains (Fig 6C). To determine whether the reduced virulence of the *hatT*::*CT* strain is due to a reduction in toxins TcdA or TcdB, we assessed toxin production in the WT, *hatR*::*CT*, and *hatT*::*CT* strains using a cell-rounding cytotoxicity assay. These data revealed toxin levels to be equivalent between all tested strains on day 4 of the infection (Fig 6D), suggesting that the reduced virulence of the *hatT*::*CT* strain *in vivo* is independent of *C*. *difficile* toxins. Taken together, these data suggest that the *hatRT* operon senses and detoxifies intracellular heme in *C*. *difficile* and is required for full pathogenicity during infection.

## Discussion

*C*. *difficile* infection of the colon causes severe epithelial cell damage, inflammation, and edema, which leads to the hallmarks of *C*. *difficile*-colitis. Importantly, this damage and subsequent inflammatory response also creates a hostile environment for bacteria within the gut [2, 31–33]. Highly reactive heme molecules that can be toxic to bacteria are released into the lumen through erythrocyte lysis and necrotic epithelial cell death [3, 5]. Despite the hazard of heme toxicity, *C*. *difficile* thrives in the colon and survives in the presence of high heme levels. Prior to this work, the mechanism by which *C*. *difficile* resists heme toxicity were unknown. Herein, we visualized the high abundance of hemoglobin during infection, serving as a proxy for heme, in the murine ceca during CDI. We identified a molecular mechanism encoded by the *hatRT* operon to sense and detoxify heme in *C*. *difficile*. HatR functions as a transcriptional repressor of the *hatRT* operon and responds to heme concentrations through direct binding of heme. HatR-heme complexes de-repress the *hatRT* operon, leading to the HatT-mediated reduction in intracellular heme concentrations, presumably through efflux. In support of these data, strains with inactivated *hatR* or *hatT* exhibited delayed growth in the presence of heme and the *hatT*::*CT* strain conferred reduced pathology in a toxin-independent manner in a mouse model of CDI.

While heme sensing and detoxification through efflux is a conserved strategy in multiple Gram-positive organisms, this report is the first to describe an obligate anaerobic pathogen containing such a system [16–18, 34]. TetR-family transcriptional regulators that bind heme have been identified, including HrtR in *Lactococcus lactis*, whereby HrtR regulates heme efflux through a system orthologous to HrtAB [17, 35]. However, HatR shares limited sequence homology (38% amino acid identity) with HrtR. Additionally, the heme binding motifs (single histidine versus two histidines) and the heme-complex disassociation constant (HatR k_d_ = 9.2 ± 1.8 μM, HrtR k_d_ = 0.4 ± 0.2 μM) differ between HatR and HrtR [17]. The significant overexpression of the *hatRT* operon in the presence of heme but not protoporphyrin IX or iron suggests the formation of the heme-HatR complex involves direct binding to the coordinated iron center of heme. The increased heme sensitivity in the *hatR*::*CT* strain despite the constitutive expression of *hatT*, suggests HatR may also function to reduce heme toxicity through sequestration. The eventual *in vitro* growth observed when the *hatT*::*CT* strain is exposed to high heme suggests the existence of other mechanisms of heme detoxification in *C*. *difficile* or the occurrence of suppressor mutations to relieve intracellular heme concentrations through a different transport system. A bioinformatics comparison of HatT with *S*. *aureus* HrtAB and the dual *S*. *agalactiae* efflux system PefAB/CD, suggests that these systems arose through convergent evolution as there is little homology between these transporters despite their important role in heme detoxification [18, 34].

The mechanisms of heme toxicity in bacteria are not completely understood. In an anaerobic environment, heme toxicity has been attributed to membrane disruption and DNA damage due to the hydrophobic structure of heme [12–14, 36]. Bilirubin, the terminal metabolite in heme catabolism in mammals is present in high concentrations in the gastrointestinal tract, and destabilizes the membrane of Gram-positive bacteria, suggesting that heme degradation products may also contribute to toxicity [37]. In *C*. *difficile*, heme enters the intracellular compartment through an unknown mechanism. It is also not known if *C*. *difficile* utilizes heme as a cofactor or metabolite. Bioinformatic analyses do not reveal heme degradation enzymes of the IsdG or HO enzyme families in *C*. *difficile* [38–40]. Additionally, it appears as if *C*. *difficile* cannot use heme as a sole iron source [41]. In this study, we demonstrated that heme accumulates in the cytoplasm of *C*. *difficile* and is subsequently detoxified through removal by HatT.

Results reported in this work demonstrate the importance of heme detoxification in CDI as the *hatT*::*CT* strain was less pathogenic in a mouse model of infection. The colonization of the WT, *hatR*::*CT*, and *hatT*::*CT* strains are at similar levels, supporting a model in which resistance to heme toxicity is important for the end stages of acute infection after serious injury to the intestinal epithelium has occurred. This observation is further supported by the reduction in disease that was only observed on days 3 and 4 following infection in the *hatT*::*CT* infected mice. The lack of phenotype of the *hatR*::*CT* strain suggests that continual expression of *hatT* in the absence of HatR in this strain is sufficient to cause full disease. Surprisingly, there were no differences in bacterial burdens at these days or differences in toxin production despite less overall pathology in the *hatT*::*CT* infected mice. This suggests *C*. *difficile* utilizes either additional heme detoxification operons or compensatory mechanisms to relieve intracellular heme stress outside of HatRT and reveals the importance of toxin-independent mechanisms of virulence. Alternatively, as *C*. *difficile* has been shown to occupy different nutritional niches during infection, and heme is heterogeneously distributed throughout the infected ceca, the heme sensitive strains may be able to maintain WT levels of colonization due to occupying niches of reduced heme concentrations at a cost of pathogenicity [42]. These results provide a molecular insight into how *C*. *difficile* adapts to the harsh environment of the inflamed gut. Further studies must be performed to elucidate additional mechanisms of protection that *C*. *difficile* utilizes to survive during infection.

## Materials and methods

### Ethics statement

All animal experiments under protocol M1700053 were reviewed and approved by the Institutional Animal Care and Use Committee of Vanderbilt University. Procedures were performed according to the institutional policies, Animal Welfare Act, NIH guidelines, and American Veterinary Medical Association guidelines on euthanasia.

### Bacterial strains, growth conditions, and plasmids

Strains used in this study are listed in S1 Table. *C*. *difficile* strains were grown at 37°C in an anaerobic chamber (85% nitrogen, 10% hydrogen, 5% carbon dioxide, Coy Lab Products) in brain-heart-infusion broth (BD Life Sciences) supplemented with 0.5% yeast extract (BD Life Sciences) and 0.1% cysteine (Sigma-Aldrich) (BHIS) or in *C*. *difficile* minimal media (CDMM) described previously [43]. *Escherichia coli* strains were grown in lysogeny broth (LB) or agar (LBA), supplemented with 50 μg/mL kanamycin or 50 μg/mL carbenicillin when necessary [43]. *Bacillus subtilis* strains were grown on LBA or in BHI broth supplemented with 5 μg/mL tetracycline or 2.5 μg/mL chloramphenicol. All antibiotics were purchased from Sigma-Aldrich.

*hatR*::*CT and hatT*::*CT strain generation*. Gene inactivations were achieved using the ClosTron system as described previously [44]. Briefly, gBlocks containing specific modifications for insertion into the genome were generated using the TargeTronics algorithm (http://www.targetrons.com) and synthesized by Integrated DNA Technologies. The gBlocks were cloned into pCR-Blunt vector using the Zero Blunt PCR cloning kit (ThermoFisher Scientific) followed by restriction digest with BsrgI and HindIII (NEB) and ligation (NEB T4 ligase) into pJS107. Plasmids were transformed into the *recA*^+^ *E*. *coli* MG1655 through a standard heat shock protocol followed by transformation into *B*. *subtilis* JH2 using an established method [44]. *B*. *subtilis* strains containing the pJS107_*hatR* or pJS107_*hatT* plasmids were mated with *C*. *difficile* R20291 overnight at 37°C by plating and mixing together 100 μL of each strain onto a BHIS plate in the anaerobic chamber. Plates were scraped and transferred into 2 mL of BHIS prior to plating 200 μL onto BHIS plates containing 20 μg/mL thiamphenicol and 50 μg/mL kanamycin (BHIS_thiamp20kan50_). Colonies from these plates were patched onto new BHIS_thiamp20kan50_ and BHIS plates containing 5 μg/mL tetracycline (BHIS_tet5_). Patched colonies that were tetracycline sensitive were patched again onto new BHIS_thiamp20kan50_ and BHIS_tet5_ plates. Colonies that remained tetracycline sensitive were streaked onto BHIS plates containing 20 μg/mL lincomycin (BHIS_linc20_). Inactivation of the *hatR* or *hatT* gene was confirmed by performing PCR to identify a 1.5 kbp shift in gene size using gDNA extracted as previously described on colonies that were lincomycin resistant [44].

*xylE reporter and complementation plasmids*. Reporter and complementation plasmids (S1 Table) were created by GenScript using the pJS116 plasmid as a backbone for the synthesized intergenic region (236 bp) of *hatR* fused to the *xylE* reporter gene, the intergenic and full coding region of *hatR*, and intergenic region of *hatR* fused to the full coding region of *hatT*. *C*. *difficile* strains were transformed as described above with the removal of the lincomycin selection and were maintained on BHIS_thiamp20_ to ensure plasmid retention.

*Protein expression plasmids*. Protein expression plasmids for HatR were generated by amplifying *hatR* flanked by BamHI and XhoI and cloning into the multiple cloning site of pLM302 after restriction digest. Point mutant generation in pLM302_*hatR* was performed with NEB Q5 Site Directed Mutagenesis kit according to the manufacturer’s instructions, using the primers listed in S2 Table. Mutations to Ala or Leu were governed by the surrounding protein motifs and retention of spatial arrangement.

*Heme toxicity growth assays*. Freshly streaked bacterial colonies were used to inoculate 5 mL of BHIS or BHIS_thiamp20_ and grown for 16 h at 37°C. Cultures were subcultured 1:50 into fresh BHIS or BHIS_thiamp20_ and grown for 6 h at 37°C prior to 1:50 inoculation into CDMM or CDMM_thiamp20_ containing heme at the indicated concentrations. All growth assays were performed in a 96-well plate in 200 μL of media. Optical density at 600 nm (OD_600_) served as measurement of growth and was measured every 30 min for the indicated total time in an EpochII microplate reader (BioTek).

### RNA extraction and sequencing

*C*. *difficile* were grown anaerobically in triplicate in CDMM in 0 or 50 μM heme. Hemin (Sigma) was solubilized in 0.1 M NaOH. The cultures were grown at 37°C to an OD_600_ of 0.3 abs. Upon reaching this density, a 1:1 solution of acetone:ethanol was added to an equal volume of the culture. Samples were stored at -80°C until used for RNA extraction. Samples were thawed on ice, pelleted, and resuspended in 750 μL of LETS buffer (1 M LiCl, 0.5 M EDTA, 1 M Tris pH 7.4). Cells were transferred to tubes containing lysing matrix B beads (MP Biomedicals) and lysed by a FastPrep-24 (MP Biomedicals) bead beater for 45 s at 6 m/s. Lysed samples were heated for 5 min at 55°C and pelleted by centrifugation for 10 min. The supernatant was transferred to a fresh tube and 1 mL TRIzol (Thermo Scientific) was added. Chloroform (200 μL) was added to each sample and vortexed prior to separation of the aqueous and organic layers by centrifugation for 15 min. The aqueous (upper) layer was transferred to a fresh tube and the RNA was precipitated through the addition of 1 mL isopropyl alcohol. Samples were incubated for 10 min and RNA was pelleted by centrifugation for 10 min. Supernatant was removed and the RNA pellet was washed with 200 μL of 70% ethanol. Samples were air dried for 1 min, then resuspended in 100 μL RNase free water. DNA contamination was removed through the addition of 8 μL RQ1 DNase, 12 μL 10x RQ1 buffer, and 2 μL RNase inhibitor (Promega) to the purified RNA. Samples were DNase treated for 2 h and purified using the RNeasy miniprep RNA cleanup kit (Qiagen). RNA concentration was determined using the Synergy 2 with Gen 5 software (BioTek).

*RNA-seq library preparation and sequencing*. RNA-seq library construction and sequencing was performed by HudsonAlpha. Concentration was determined using the Quant-iT RiboGreen RNA assay (Thermo Scientific) and integrity was visualized using an RNA 6000 nano chip (Agilent) on an Agilent 2100 Bioanalyzer (Applied Biosystems). RNA was normalized to 500 ng of total RNA for each sample and the ribosomal RNA (rRNA) was removed using Ribo-Zero rRNA Removal Kit (Illumina). Directly after rRNA removal, the RNA was fragmented and primed for first strand synthesis using the NEBNext First Strand synthesis module (New England BioLabs Inc.) followed by second strand synthesis using NEBNext Ultra Directional Second Strand synthesis kit. Library preparation was achieved using NEBNext DNA Library Prep Master Mix set for Illumina with minor modifications. PolyA addition and custom adapter ligation was performed following end–repair. Post-ligated samples were individually barcoded with unique in-house Genomic Services Lab (GSL) primers and amplified through 12 cycles of PCR. Library quantity was assessed by Qubit 2.0 Fluorometer (Invitrogen), and quality was determined using a DNA High Sense chip on a Caliper Gx (Perkin Elmer). Final quantification of the complete libraries for sequencing applications was measured using the qPCR-based KAPA Biosystems Library Quantification kit (Kapa Biosystems, Inc.). Libraries were diluted to 12.5 nM and pooled equimolar prior to clustering. Paired-End (PE) sequencing was performed on an Illumina HiSeq2500 sequencer (Illumina, Inc.). Raw sequence data are deposited on the NCBI Sequence Read Archive.

*Processing of RNA-seq reads*. RNA-seq analysis was performed by HudsonAlpha utilizing their unique in-house pipeline. Briefly, quality control was performed on raw sequence data from each sample using FastQC (Babraham Bioinformatics). Curated raw reads were imported into the data analysis platform, Avadis NGS (Strand Scientifics) and mapped to the reference *C*. *difficile* R20291 genome. Aligned reads were filtered on various criteria to ensure the highest read quality. Replicate samples were grouped and quantification of transcript performed using Trimmed Means of M-values (TMM) as the normalization method. Differential expression of genes was calculated using fold change (using default cut-off ≥ ±2.0) observed between conditions, and the p-value of the differentially expressed gene list was estimated by Z-score calculations using determined by Benjamini Hochberg FDR correction of 0.05 [45]. The genome alignment figure (Fig 2A) was created using Circos with a max of 30,000 RPKM displayed.

### Quantitative RT-PCR

RNA was extracted as described above and 2 μg was reverse transcribed by M-MLV reverse transcriptase (Fisher Scientific) in the presence of RNase inhibitor (Promega) and random hexamers (Promega). Reactions lacking the reverse transcriptase were used to control for DNA contamination. Newly created cDNA was diluted 1:100 and was used in qRT-PCR using iQ SYBR green supermix (BIO-RAD) utilizing the primer pairs in S2 Table. Amplification was achieved using a 3-step melt cure program on a CFX96 qPCR cycler (BIO-RAD). Transcript abundance was calculated using the ΔΔCT method normalized by the *rpoB* gene.

### Polyclonal antibody generation

HatR was purified as described below and fresh protein was submitted to the Vanderbilt Antibody and Protein Resource core for generation of a rabbit polyclonal antibody against HatR. This antibody was affinity purified for increased HatR specificity. The α-HatR antibody was tested for specificity and reactivity in immunoblot analysis of purified HatR protein in addition to whole cell lysates from heme treated WT and *hatR*::*CT* strains.

### Immunoblotting analysis

WT or *hatR*::*CT* strains were grown in 5 mL of BHIS overnight at 37°C. Cultures were subcultured into fresh BHIS containing 0, 1, 5, 10 or 25 μM heme and grown for 6 h. Cells were pelleted by centrifugation (4000 x *g* for 10 min), supernatant was removed and were resuspended in 1 mL of 1 X PBS containing 2.5 mg/mL lysozyme (ThermoFisher Scientific). Samples were incubated for 1 h at 37°C, pelleted by centrifugation (20,000 x *g* for 5 min), then resuspended in 1 X PBS followed by sonication using Ultrasonic dismembrator (ThermoFisher Scientific) to lyse the cells. Debris from the lysed cells was pelleted by centrifugation (20,000 x *g* for 5 min). Supernatant was used in immunoblotting analysis using rabbit polyclonal α-HatR antibodies as previously described [46]. Detection was performed using a goat anti-rabbit IgG (H+L) cross-adsorbed secondary antibody with an Alexa Fluor 680 and imaged using a ChemiDoc MP imaging system (Bio-Rad).

### Protein expression and purification

*E*. *coli* BL21 (DE3) pREL containing the pML302_*hatR* plasmids were grown overnight in 5 mL of LB_kan50_ at 37°C. Cells were subcultured into Terrific broth (ThermoFisher Scientific) containing 50 μg/mL kanamycin and grown to the mid-logarithmic phase of growth (0.5 abs measured at 600 nm) at 37°C prior the addition of 1 mM isopropyl-1-thiol-D-galactopyranoside (IPTG). Growth was continued at 16°C for 16 h. Cells were harvested by centrifugation (6000 x *g* for 10 min) and resuspended in 1 X PBS. Cells were lysed by passage through an EmulsiFlex homogenizer (Avestin) three times at 20,000 lb/in^2^. The insoluble debris was removed by centrifugation at 40,000 x *g* for 1 h and the supernatant was filtered using a 0.22-μM-pore sizer filter. Filtered lysate was added to amylose resin (New England Biolabs Inc.) and allowed to bind at 4°C for 30 min prior to transfer to a gravity column. The column was washed with four column volumes of wash buffer (20 mM Tris-HCl, 500 mM NaCl, 1 mM EDTA, pH 7.5) three times followed by 2 column volumes of elution buffer (20 mM Tris-HCl, 500 mM NaCl, 1 mM EDTA, 10 mM maltose, pH 7.5) twice. The maltose-binding protein tag (MBP) was cleaved using the Pierce HRV 3C Protease Solution kit (ThermoFisher) by following the manufacturer’s instructions. Cleaved tag and protease were removed by the addition of HisPur Cobalt Resin (ThermoFisher) and allowed to bind at 4°C for 1 h with rotation. Beads were pelleted by centrifugation (2000 x *g* for 2 min) and the supernatant containing tagless protein was removed.

### Absorption spectroscopy

Heme binding by HatR were determined by measuring the absorption spectrum of increasing amounts of hemin (0–25 μM) after addition to a cuvette containing 10 μM recombinant HatR in 1 mL of Tris-buffered saline (TBS) and a reference standard containing 1 mL TBS on a Varian Cary 50BIO. Samples were mixed and allowed to incubate at room temperature in the dark for 5 min prior to collecting the spectrum between 300–800 nm with 10 nm increments. Binding ratio of heme to HatR was determined by plotting the change in absorbance at 413 nm between the reference standard and the HatR sample. A curve fit and ratio was obtained by performing the one-site binding model non-linear regression function on Graph Pad Prism 6.

### XylE reporter assays

Bacteria harboring the reporter plasmid pJS116_*phatR-xylE* were grown overnight in BHIS_thiamp20_ and subcultured 1:50 into 10 mLs of fresh BHIS_thiamp20_ containing 0 or 10 μM heme. Cultures were grown for 6 h at 37°C prior to cytoplasmic fraction preparation and analysis of XylE activity as described previously [16]. Absolute XylE activities were determined spectrophotometrically by measuring the formation of 2-hydroxymoconic acid from catechol for *C*. *difficile* reporters due to lysozyme interference during protein quantification.

### LC-MS heme quantification

#### Bacterial growth

*C*. *difficile* WT and *hatT*::*CT* strains were streaked onto BHIS and grown for 16 h at 37° C. Single colonies were used to inoculate 5 mL cultures in BHIS and grown for 16 h at 37° C. Seven hundred fifty μL of these cultures were subcultured into 75 mL of BHIS and BHIS + 25 μM heme in an Erlenmeyer flask and grown for 16 h at 37° C. Total CFU were determined by serial dilution and plating onto BHIS for enumeration, and cells were collected by centrifugation and flash frozen in liquid nitrogen before storage at -80°C.

#### Preparation of standard curves

A 2 mM stock solution of a heme standard was prepared in DMSO. This stock was then diluted to make standards from 0.25–6 μM in acetonitrile + 0.1% trifluoracetic acid (TFA).

#### Extraction of heme

*C*. *difficile* cell pellets were thawed on ice. 1 mL of 1M HCl:DMSO (1:1, v/v) was added and samples were vortexed. Samples were transferred to 2 mL FastPrep lysis B matrix tubes and the cells lysed by bead beating in a FastPep-24 5G instrument (6.0 m/sec, 40 s total, 2X). The cell lysate was centrifuged to pellet debris (10,000 rpm, 5 min, 4°C). Supernatants were collected and kept in the dark. The pellets were resuspended in 1 mL of 1M HCl:DMSO (1:1, v/v), vortexed vigorously, and centrifuged again as above. The supernatants were pooled and the resuspension/centrifugation cycle repeated one more time. The pooled supernatants were filtered using a 0.22 μm Millex-GS syringe filter (MF-Millipore) and subsequently diluted to 25 mL with ddH2O. Using a Sep-Pak Vac 3cc tC18 cartridge (Waters 036815), the extracts were concentrated and subsequently eluted with 2 mL of acetonitrile + 0.1% TFA then 2 mL of methanol. Extracted porphyrins were dried under N2 (g) and resuspended in 100 μL of acetonitrile + 0.1% TFA. All samples were immediately dispensed into vials for mass spec analysis.

#### LC-MS analysis

Samples were prepared by adding 25 μL of ultrapure water to 75 μL solutions of analytes in acetonitrile + 0.1% TFA. A PLRP-S column (Agilent) was equilibrated to an 85:15 ratio of solvent A (ultrapure water + 0.1% formic acid) to solvent B (acetonitrile + 0.1% formic acid). Liquid chromatography separations were achieved by linear gradient elution, transitioning from 15% to 95% solvent B over 6 min followed by a 2 min hold at 95% B. The column was re-equilibrated to 15% solvent B for 2 min in between injections of the same sample (two technical replicates run per sample, 2 μL injection volume, 600 μL/min flow rate, 50°C). Two blank runs were implemented between samples to ensure against column holdover of analytes. Electrospray ionization mass spectrometry analysis was carried out in positive mode with a capillary voltage of 2Hz (Agilent 6538 UHD Q-TOF).

#### Quantification of standards and analytes from LC-MS data

Data were analyzed using MassHunter Qualitative Analysis Software and MZmine 2 [47]. Extracted ion chromatograms (EICs) were derived for each individual standard on the basis of its mass per charge (*m/z*) in positive ion mode, which is equivalent to the exact mass of its positive ion (M+H)^+^. Values for *m/z* were determined empirically for all standards. Peaks associated with each analyte were integrated. For the generation of standard curves, integrated peak areas were plotted versus concentration. Linear regression analysis (Kaleidagraph) was used to determine the correlation coefficient between integrated peak area and heme concentration (slope of standard curve, mporph). For the quantification of analytes from cells, values for the integrated peak intensities (measured in units of ion counts) were converted to units of concentration (μmol/L injected) via: counts x (mporph)^-1^. The concentration of each analyte in the injected volumes [A] was subsequently converted to units of nmol analyte per CFU in sample as: [A] x (volume used to resuspend dried sample) x (CFUs in analyzed cell pellet)^-1^. Reported values are averages of 3 biological samples and two technical replicates.

### Mouse model of CDI

Adult (8–12 week old) age-matched male C57Bl/6 (Jackson Laboratories) were housed in groups of five and maintained at Vanderbilt University Medical Center Animal Facilities. Mice were subjected to a previously described model of CDI [24, 27]. Briefly, mice were treated with 0.5 mg/mL cefoperazone in their drinking water for 5 days. Mice were given a 2 day recovery period prior to administration of 10^5^ spores of WT, *hatR*::*CT*, or *hatT*::*CT C*. *difficile* strains in PBS via oral gavage. Prior to infection, mice were confirmed to be *C*. *difficile* negative. After infection, mice were monitored for signs of disease, including diarrhea and weight loss. Mice that displayed severe disease or weight loss greater than 20% were humanely euthanized.

#### Bacterial burden determination

*C*. *difficile* CFUs were quantified daily from fecal samples. Samples were diluted and homogenized in PBS and serial plated onto taurocholate cycloserine cefoxitin fructose agar (TCCFA) for enumeration as CFU per gram of feces.

#### Histological analysis

On the final day of infection and necropsy, ceca were harvested, fixed in a 10% formalin solution and embedded in paraffin. Cut sections were stained with hematoxylin and eosin (H&E). Stained sections were assigned a disease score in a blinded fashion by a pathologist based on previously established criteria [48]. Histological scores are presented as a sum of three independent criteria: epithelial damage, edema, and inflammation.

#### Imaging mass spectrometry

MALDI IMS was performed as previously described [25, 26]. Briefly, ceca were harvested after necropsy and flash frozen in liquid nitrogen in a 25% Optimal Cutting Temperature compound. Sections were sequentially washed to remove interfering lipids, salts, and OCT using 70% ethanol for thirty seconds, 100% ethanol for thirty seconds, 6:2:1 ethanol:chloroform:acetic acid for 2 minutes, 70% ethanol for thirty seconds, and 100% ethanol for thirty seconds. Slides were dried in a desiccator before MALDI matrix was applied. Fifteen mg/mL 2,5-dihydroxyacetophenone was prepared in 90% acetonitrile with 0.2% TFA and crystals were dissolved by sonication for ten minutes. Matrix was applied six times using a TM-Sprayer (HTX Imaging) operated at 1100 mm/min and at a flow rate of 0.2 mL/min using 90% acetonitrile as a pushing solvent. The spray nozzle was heated to 80°C with the track spacing set to 2 mm. Coating was rehydrated using 1 mL of 50 mM acetic acid in a sealed hydration chamber for 3 min at 85°C. IMS was performed using a rapifleX MALDI Tissuetyper (Bruker Daltonics) operated in linear positive ion mode. The laser was operated at 10,000 hertz in single mode and pixels were set to be 50 by 50 μm. A total of five-hundred laser shots were captured per pixel with fifty laser shots at each position within the pixel. Data were processed using fleXimaging version 4.1. Data were further analyzed using SCiLS Lab 2015b version 3.02.7774 (Bruker Daltonics). Spectra were normalized to total ion count and baseline subtracted using a top hat algorithm. The images display the ion map of the m/z value of 14,995 without denoising but with interpolation turned on.

### *C*. *difficile* toxin cytotoxicity determination

Green African monkey kidney epithelial (Vero, ATCC CCL-81) cell-rounding cytotoxicity assays were performed as previously described [27]. Cells were grown to confluence in Dulbecco modified Eagle medium (DMEM, Gibco Laboratories) with 1% penicillin-streptomycin (Gibco Laboratories) and 10% fetal bovine serum (Gibco Laboratories) prior to plating at a total cell density of 10^5^ cells per well in a 96-well plate. Fresh fecal samples were normalized to weight, diluted and homogenized in sterile PBS. Fecal debris was pelleted by centrifugation (13,000 x *g* for 5 min) and tenfold serial dilutions of supernatants were added to the wells of Vero cells. Complete cell-rounding for each dilution was assessed after overnight incubation at 37°C with 5% CO_2_. Confirmation of *C*. *difficile* toxin A and toxin B were achieved by neutralization of cell rounding with a combined antitoxin antisera (Techlab). Cell rounding cytotoxicity titers are presented as the log_10_ of the reciprocal value of the highest dilution with complete rounding of cells.

### Statistical analysis

All data analysis and statistical tests were performed in GraphPad Prism X software. Specific statistical tests, replicate numbers, calculated errors and other information for each experiment are reported in the figure legends.

## Supporting information

S1 Fig Additional images of hemoglobin accumulation in the cecum during *C. difficile* infection. (A) H&E images of *C*. *difficile* strain R20291 infected C57BL/6 mice. (B) Abundance of hemoglobin subunit alpha in serial sections of the same ceca determined by MALDI IMS. Scale bars, 5 mm. Each image pair is an independent ceca from a distinct mouse.
(TIF)

S2 Fig Complementation of *hatR::CT* and *hatT::CT* heme sensitivity. Growth of *C*. *difficile* WT pJS116 (empty vector), *hatR*::*CT* pJS116, *hatR*::*CT* pJS116_*phatR-hatR*, *hatT*::*CT* pJS116, and *hatT*::*CT* pJS116_*phatR-hatT* strains in CDMM in the presence or absence of heme (50 μM). The data are a representative from three independent experiments each in biological triplicate with standard error of the mean. μM refers to concentration of heme.
(TIF)

S3 Fig Histidine residues within HatR that are not required for heme binding. Absorption spectra of 10 μM heme binding to 10 μM HatR, HatR H121A (A), HatR H126L (B), HatR H165A (C), and HatR H180A (D).
(TIF)

S4 Fig Purified recombinant HatR and HatR H99L. Coomassie stained SDS-PAGE of purified recombinant HatR and HatR H99L. 1 = protein ladder. 2 = HatR (22 kDa). 3 = HatR H99L (22 kDa).
(TIF)

S1 Table Bacterial strains and plasmids used in this study. (DOCX)

S2 Table Oligonucleotides used in this study. (DOCX)

S3 Table *C. difficile* R20291 genes transcriptionally up-regulated in the presence of heme. (DOCX)

S4 Table *C. difficile* R20291 genes transcriptionally down-regulated in the presence of heme. (DOCX)
