# Supplementary figures and images for: Heme sensing and detoxification by HatRT contributes to pathogenesis during Clostridium difficile infection
Source: PLoS Pathog. 2018 Dec 21;14(12):e1007486. doi: 10.1371/journal.ppat.1007486 (PMC6303022; doi:10.1371/journal.ppat.1007486)

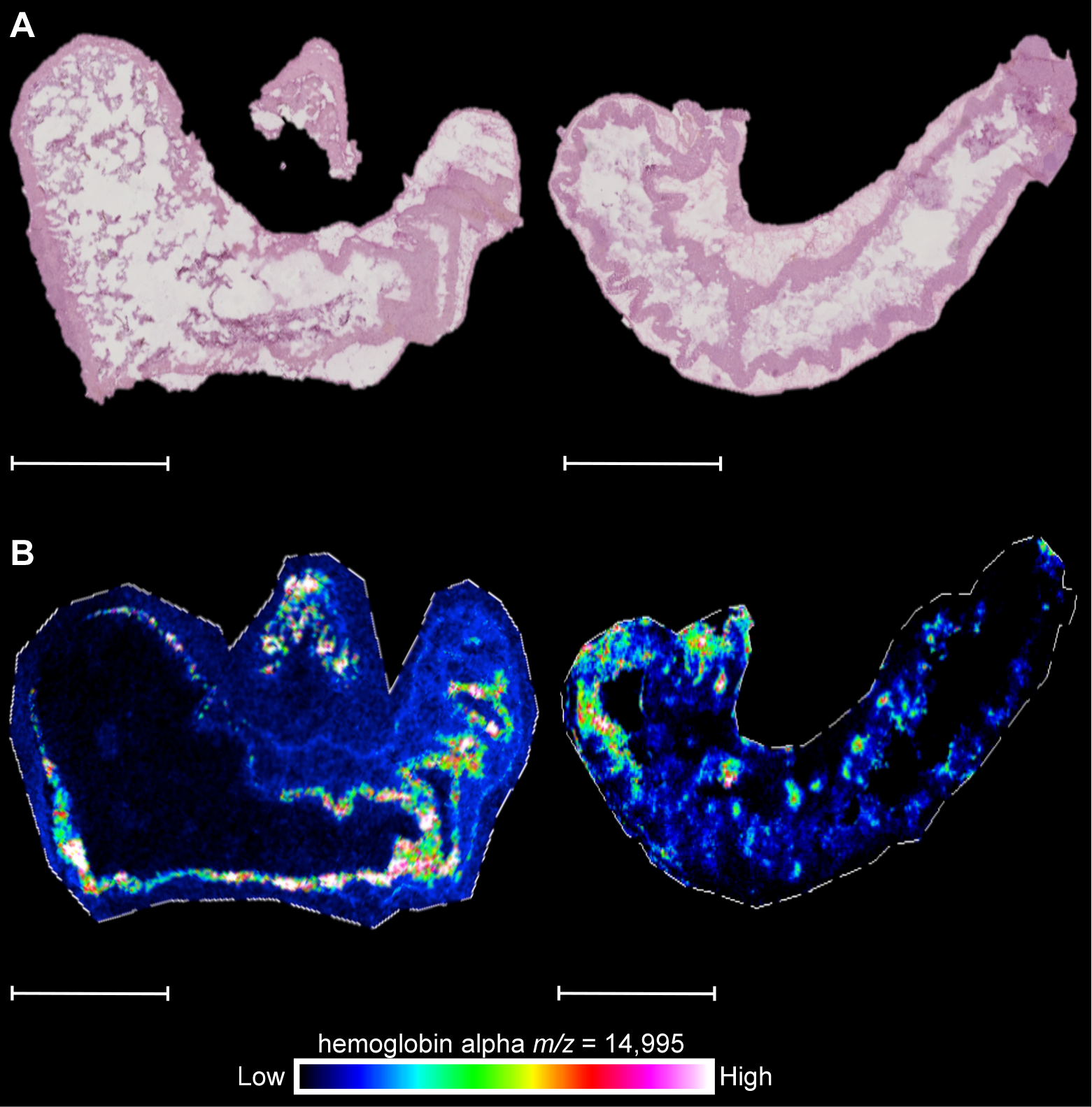

Supplement: S1 Fig — (A) H&E images of C. difficile strain R20291 infected C57BL/6 mice. (B) Abundance of hemoglobin subunit alpha in serial sections of the same ceca determined by MALDI IMS. Scale bars, 5 mm. Each image pair is an independent ceca from a distinct mouse. (TIF) [file ppat.1007486.s001.tif]

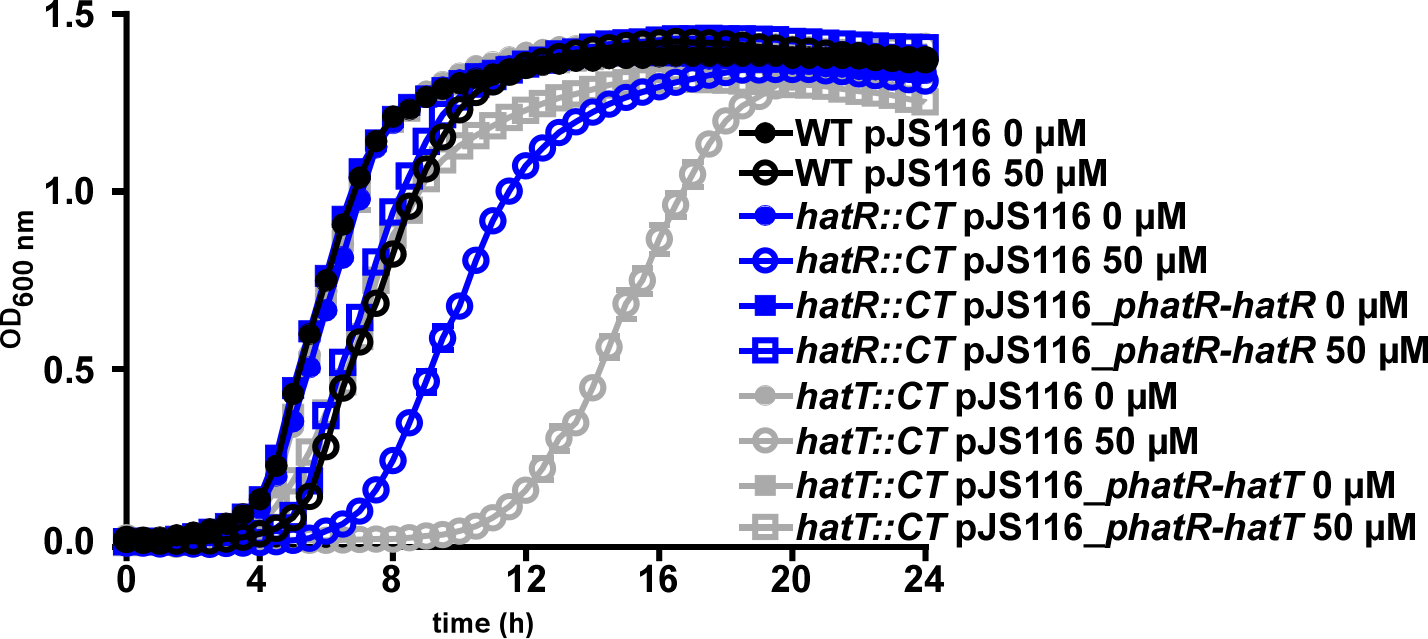

Supplement: S2 Fig — Growth of C. difficile WT pJS116 (empty vector), hatR::CT pJS116, hatR::CT pJS116_phatR-hatR, hatT::CT pJS116, and hatT::CT pJS116_phatR-hatT strains in CDMM in the presence or absence of heme (50 μM). The data are a representative from three independent experiments each in biological triplicate with standard error of the mean. μM refers to concentration of heme. (TIF) [file ppat.1007486.s002.tif]

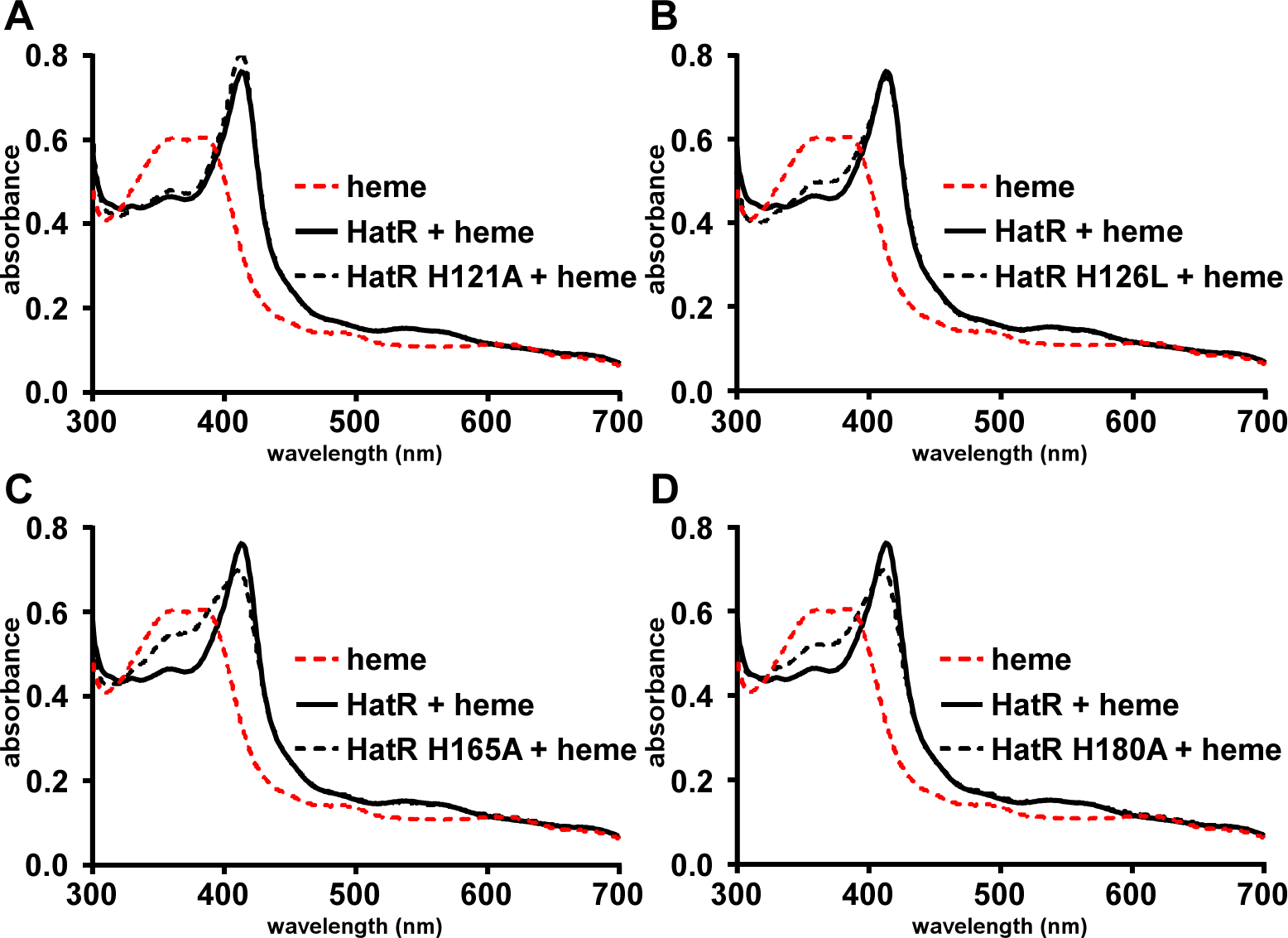

Supplement: S3 Fig — Absorption spectra of 10 μM heme binding to 10 μM HatR, HatR H121A (A), HatR H126L (B), HatR H165A (C), and HatR H180A (D). (TIF) [file ppat.1007486.s003.tif]

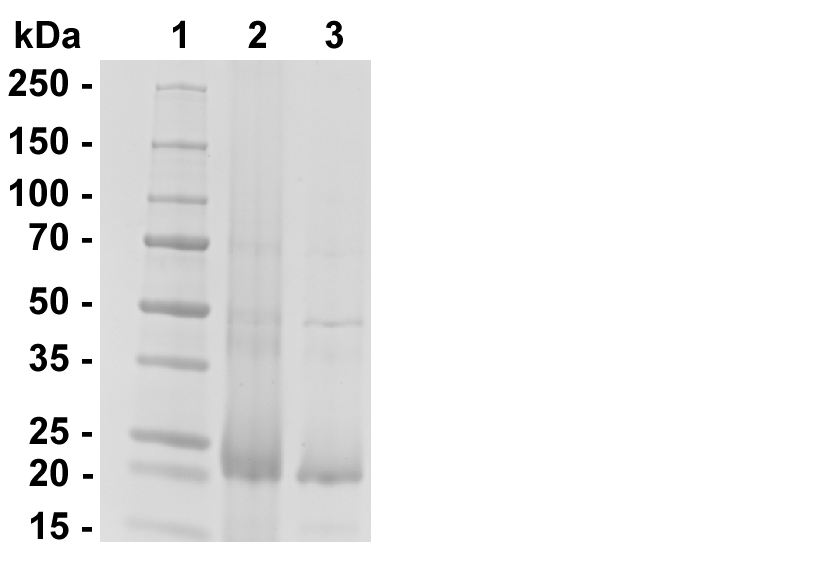

Supplement: S4 Fig — Coomassie stained SDS-PAGE of purified recombinant HatR and HatR H99L. 1 = protein ladder. 2 = HatR (22 kDa). 3 = HatR H99L (22 kDa). (TIF) [file ppat.1007486.s004.tif]
