# Supplementary material for: Heme sensing and detoxification by HatRT contributes to pathogenesis during Clostridium difficile infection
Source: PLoS Pathog. 2018 Dec 21;14(12):e1007486. doi: 10.1371/journal.ppat.1007486 (PMC6303022; doi:10.1371/journal.ppat.1007486)
Supplement: S1 Table — (DOCX) [file ppat.1007486.s005.docx]

**S1 Table. Bacterial strains and plasmids used in this study.**

| **Bacterial Strain or plasmid** | **Relevant Feature or Genotype** | **Reference** |
| --- | --- | --- |
| *Clostridium difficile* R20291 |  | [1] |
| *Clostridium difficile hatR::CT* | Intron inserted into *hatR* | This study |
| *Clostridium difficile hatT::CT* | Intron inserted into *hatT* | This study |
| *Bacillus subtilis* JH BS2 | Carries Tn196 | [2] |
| *Escherichia coli* DH5α |  | [3] |
| *Escherichia coli* MG1655 | RecA+ | [4] |
| *Escherichia coli* BL21(DE3) |  | [5] |
| pJS107 | ClosTron plasmid | [2] |
| pJS107_*hatR* | ClosTron plasmid with intron targeted to *hatR* | This study |
| pJS107_*hatT* | ClosTron plasmid with intron targeted to *hatT* | This study |
| pJS116 | Stable *C. difficile* plasmid | [2] |
| pJS116_*phatR-hatR* | *hatR::CT* complementation plasmid | This study |
| pJS116_*phatR-hatT* | *hatT::CT* complementation plasmid | This study |
| pJS116_*phatR-xylE* | XylE reporter gene driven by the promoter of *hatR* | This study |
| pLM302 | Protein expression plasmid | Center for Structural Biology, Vanderbilt University |
| pLM302_*hatR* | HatR expression plasmid | This study |
| pLM302_*hatR-*H99L | HatR-H99L expression plasmid | This study |
| pLM302_*hatR-*H121A | HatR-H121A expression plasmid | This study |
| pLM302_*hatR-*H126L | HatR-H126L expression plasmid | This study |
| pLM302_*hatR-*H165A | HatR-H165A expression plasmid | This study |
| pLM302_*hatR-*H180A | HatR-H180A expression plasmid | This study |

1. Stabler RA, He M, Dawson L, Martin M, Valiente E, Corton C, et al. Comparative genome and phenotypic analysis of *Clostridium difficil*e 027 strains provides insight into the evolution of a hypervirulent bacterium. Genome Biol. 200910(9):R102. doi: 10.1186/gb-2009-10-9-r102. PubMed PMID: 19781061 PubMed Central PMCID: PMCPMC2768977.

2. Francis MB, Allen CA, Shrestha R, Sorg JA. Bile acid recognition by the *Clostridium difficile* germinant receptor, CspC, is important for establishing infection. PLoS Pathog. 20139(5):e1003356. doi: 10.1371/journal.ppat.1003356. PubMed PMID: 23675301 PubMed Central PMCID: PMCPMC3649964.

3. Hanahan D. Studies on transformation of *Escherichia coli* with plasmids. J Mol Biol. 1983166(4):557-80. PubMed PMID: 6345791.

4. Blattner FR, Plunkett G, Bloch CA, Perna NT, Burland V, Riley M, et al. The complete genome sequence of *Escherichia coli* K-12. Science. 1997277(5331):1453-62. PubMed PMID: 9278503.

5. Jeong H, Barbe V, Lee CH, Vallenet D, Yu DS, Choi SH, et al. Genome sequences of *Escherichia coli* B strains REL606 and BL21(DE3). J Mol Biol. 2009394(4):644-52. Epub 2009/09/26. doi: 10.1016/j.jmb.2009.09.052. PubMed PMID: 19786035.
