# Supplementary material for: Heme sensing and detoxification by HatRT contributes to pathogenesis during Clostridium difficile infection
Source: PLoS Pathog. 2018 Dec 21;14(12):e1007486. doi: 10.1371/journal.ppat.1007486 (PMC6303022; doi:10.1371/journal.ppat.1007486)
Supplement: S2 Table — (DOCX) [file ppat.1007486.s006.docx]

**S2 Table. Oligonucleotides used in this study.**

| **Name** | **Sequence (5’ – 3’)** | **Description** |
| --- | --- | --- |
| qRT_*hatR*_F | ggaaaatagctcaaaggtgtgg | qRT-PCR forward primer for *hatR* |
| qRT_*hatR*_R | cgccttatcaataaatcccattc | qRT-PCR reverse primer for *hatR* |
| qRT_*hatT*_F | ttggaaagccctcaactcc | qRT-PCR forward primer for *hatT* |
| qRT_*hatT*_R | ttttgccatttcagggtctg | qRT-PCR reverse primer for *hatT* |
| qRT_*rpoB*_F | tgctgttgaaatggttcctg | qRT-PCR housekeeping gene forward primer |
| qRT_*rpoB*_R | cggttggcatcatcattttc | qRT-PCR housekeeping gene reverse primer |
| R20291_*hatR*_F | aggtgtggtataagtgcaggt | Forward primer to check for intron insertion into *hatR* |
| R20291_*hatR*_R | agctgttcatgaaagtcgtc | Reverse primer to check for intron insertion into *hatR* |
| R20291_*hatT*_F | gtggtgtttaccttgaatcataat | Forward primer to check for intron insertion into *hatT* |
| R20291_*hatT*_R | cttgaacctaaaatattggcaatacc | Reverse primer to check for intron insertion into *hatT* |
| *hatR*_BamHI_F | ggggatccgggatgccaaagattttagaaaatg | Forward primer for cloning *hatR* into pLM302 |
| *hatR*_XhoI_R | gggctcgaggggttaatgtattaatttttcaata | Reverse primer for cloning *hatR* into pLM302 |
| *hatR*_H99L_F | gaaattttataaacaaagttcttaatatccaatacaatg | Forward primer for H99L point mutation in *hatR* |
| *hatR*_H99L_R | cattgtattggatattaagaactttgtttataaaatttc | Reverse primer for H99L point mutation in *hatR* |
| *hatR*_H121A_F | gaaattttataaacaaagttcttaatatccaatacaatg | Forward primer for H121A point mutation in *hatR* |
| *hatR*_H121A_R | cattgtattggatattaagaactttgtttataaaatttc | Reverse primer for H121A point mutation in *hatR* |
| *hatR*_H126L_F | caaagacgactttcttgaacagctttcaaataaag | Forward primer for H126L point mutation in *hatR* |
| *hatR*_H126L_R | ctttatttgaaagctgttcaagaaagtcgtctttg | Reverse primer for H126L point mutation in *hatR* |
| *hatR*_H165A_F | caacatatcttgctatagaatttgaag | Forward primer for H165A point mutation in *hatR* |
| *hatR*_H165A_R | cttcaaattctatagcaagatatgttg | Reverse primer for H165A point mutation in *hatR* |
| *hatR*_H180A_F | gaaaaattaatagcttaacccctcgag | Forward primer for H180A point mutation in *hatR* |
| *hatR*_H180A_R | ctcgaggggttaagctattaatttttc | Forward primer for H180A point mutation in *hatR* |
