# Supplementary material for: Heme sensing and detoxification by HatRT contributes to pathogenesis during Clostridium difficile infection
Source: PLoS Pathog. 2018 Dec 21;14(12):e1007486. doi: 10.1371/journal.ppat.1007486 (PMC6303022; doi:10.1371/journal.ppat.1007486)
Supplement: S3 Table — (DOCX) [file ppat.1007486.s007.docx]

**S3 Table. *C. difficile* R20291 genes transcriptionally upregulated in the presence of heme.**

| **Gene Symbol** | **Fold-change Heme Treated vs Untreated Media Control** | **Description** |
| --- | --- | --- |
| CDR20291_0781 | 31.78261 | putative membrane protein |
| CDR20291_0782 | 26.463398 | MarR-family transcriptional regulator |
| CDR20291_1227 (*hatR)* | 11.18395 | TetR-family transcriptional regulator |
| CDR20291_2306 | 6.155928 | conserved hypothetical protein |
| CDR20291_1226 (*hatT)* | 5.054025 | putative transporter |
| *tcdD* | 4.6314363 | putative transcriptional regulator |
| *ctfA* | 4.446509 | butyrate-acetoacetate CoA-transferase subunit A |
| CDR20291_0433 | 4.170941 | putative sugar-phosphate dehydrogenase |
| CDR20291_2321 | 3.9144623 | putative oxidoreductase ferredoxin subunit |
| CDR20291_1223 | 3.6944113 | putative phage regulatory protein |
| *oraS* | 3.594828 | D-ornithine aminomutase S component |
| *thlA2* | 3.492519 | putative acetyl-CoA acetyltransferase |
| CDR20291_2801 | 3.4535594 | putative membrane protein |
| CDR20291_0163 | 3.4244125 | hypothetical protein |
| CDR20291_0284 | 3.4145832 | conserved hypothetical protein |
| CDR20291_3277 | 3.3464322 | putative exported protein |
| CDR20291_1726 | 3.3368134 | hypothetical protein |
| CDR20291_0391 | 3.3224375 | putative amino acid racemase |
| *acpP* | 3.1869857 | acyl carrier protein |
| CDR20291_1554 | 3.136697 | putative membrane protein |
| CDR20291_3464 | 3.0761995 | conjugative transposon protein |
| CDR20291_1445 | 3.0670178 | hypothetical phage protein |
| CDR20291_0033 | 3.0560584 | putative membrane protein |
| CDR20291_2677 | 3.034006 | putative cell wall teichoic acid glycosylation protein |
| CDR20291_0347 | 3.0311313 | hypothetical protein |
| *spoIIIAG* | 3.0204601 | stage iii sporulation protein ag flags: precursor |
| CDR20291_3191 | 3.0179818 | conserved hypothetical protein |
| CDR20291_0738 | 2.9525864 | hypothetical protein |
| CDR20291_0739 | 2.9086928 | putative membrane protein |
| CDR20291_1810 | 2.8909168 | hypothetical protein |
| CDR20291_0291 | 2.8904557 | PTS system, IIc component |
| CDR20291_2194 | 2.8736017 | putative membrane protein precursor |
| CDR20291_0141 | 2.850998 | putative RNA-binding protein |
| *potD* | 2.8405726 | spermidine/putrescine ABC transporter, substrate-binding lipoprotein |
| CDR20291_2335 | 2.8380623 | conserved hypothetical protein |
| CDR20291_2575 | 2.8209536 | hypothetical protein |
| *u1* | 2.8027954 | putative regulatory protein |
| CDR20291_1667 | 2.77625 | hypothetical protein |
| CDR20291_1393 | 2.7436821 | hypothetical protein |
| CDR20291_0398 | 2.7361345 | putative membrane protein |
| CDR20291_3511 | 2.7244775 | putative peptidase |
| CDR20291_0728 | 2.7207413 | putative hydroxymethylglutaryl-CoA lyase |
| CDR20291_0214 | 2.7207377 | putative nitroreductase |
| CDR20291_2039 | 2.7174134 | conserved hypothetical protein |
| CDR20291_2727 | 2.6998487 | putative peptidase |
| CDR20291_2190 | 2.6926663 | putative regulatory protein |
| CDR20291_2517 | 2.6859193 | putative transcriptional regulator |
| CDR20291_3465 | 2.6800773 | conjugative transposon protein |
| CDR20291_1866 | 2.6749303 | conserved hypothetical protein |
| CDR20291_1658 | 2.6748435 | putative membrane protein |
| CDR20291_0390 | 2.6627486 | putative component of D-ornithine aminomutase |
| CDR20291_3094 | 2.6538692 | hypothetical protein |
| CDR20291_2698 | 2.6518354 | conserved hypothetical protein |
| CDR20291_1823 | 2.6514072 | putative lipoprotein signal peptidase |
| CDR20291_3453 | 2.649938 | putative collagen-binding surface protein |
| CDR20291_1078 | 2.6334527 | hypothetical protein |
| CDR20291_3460 | 2.6278205 | conjugative transposon protein |
| CDR20291_1073 | 2.6088905 | hypothetical protein |
| CDR20291_2728 | 2.6057155 | putative membrane protein |
| CDR20291_3466 | 2.5999343 | putative cell wall hydrolase |
| CDR20291_0587 | 2.592949 | hypothetical protein |
| *pgmB* | 2.5752125 | beta-phosphoglucomutase |
| CDR20291_2189 | 2.5734594 | putative repressor |
| *ydiB* | 2.5728512 | NAD-dependent shikimate 5-dehydrogenase |
| CDR20291_0288 | 2.5664334 | PTS system, IIb component |
| CDR20291_3140 | 2.555809 | PTS system, IIb component |
| CDR20291_1971 | 2.5507545 | hypothetical protein |
| CDR20291_0294 | 2.5414681 | putative peptidase |
| *rpmH* | 2.5404537 | 50S ribosomal protein L34 |
| CDR20291_1083 | 2.5396543 | putative membrane protein |
| CDR20291_3153 | 2.529364 | putative membrane protein |
| CDR20291_1319 | 2.5218627 | putative phage shock protein |
| CDR20291_3463 | 2.5192652 | conjugative tranposon protein |
| *fdxA* | 2.517662 | ferredoxin |
| CDR20291_3132 | 2.504153 | hypothetical protein |
| CDR20291_0409 | 2.501609 | putative hydrolase |
| CDR20291_1688 | 2.4785788 | putative membrane protein |
| CDR20291_1026 | 2.4770615 | putative glutamine amidotransferase |
| CDR20291_2775 | 2.476111 | hypothetical protein |
| CDR20291_1820 | 2.4603844 | hypothetical protein |
| CDR20291_3053 | 2.4462643 | putative phage-related protein |
| *rpsO* | 2.431163 | 30S ribosomal protein S15 |
| CDR20291_1192 | 2.419036 | putative lantibiotic ABC transporter, permease protein |
| CDR20291_0203 | 2.4166424 | conserved hypothetical protein |
| CDR20291_3113 | 2.415126 | two-component response regulator |
| CDR20291_2019 | 2.412923 | hypothetical protein |
| CDR20291_1222 | 2.409677 | putative phage regulatory protein |
| CDR20291_1430 | 2.4094286 | hypothetical protein |
| CDR20291_1863 | 2.4049656 | putative membrane protein |
| CDR20291_1156 | 2.4041753 | hypothetical protein |
| *spoVG* | 2.4039543 | stage V sporulation protein G |
| CDR20291_1618 | 2.4028168 | conserved hypothetical protein |
| CDR20291_2405 | 2.4002483 | putative translation inhibitor endoribonuclease |
| CDR20291_1576 | 2.3943622 | hypothetical protein (pseudogene) |
| *secG* | 2.393486 | putative subunit of preprotein translocase |
| CDR20291_3505 | 2.3905718 | GntR-family transcriptional regulator |
| CDR20291_2807 | 2.3885088 | hypothetical protein |
| *phnA* | 2.3848896 | putative phosphonoacetate hydrolase |
| CDR20291_2953 | 2.384776 | putative membrane protein |
| CDR20291_1949 | 2.3762958 | putative uncharacterized protein |
| CDR20291_3187A | 2.3746626 | autoinducer prepeptide |
| CDR20291_2396 | 2.372154 | putative D-alanyl-D-alanine carboxypeptidase |
| *sat* | 2.36493 | streptogramin A acetyltransferase |
| *rpmE* | 2.359248 | 50S ribosomal protein L31 |
| CDR20291_1126 | 2.3415961 | conserved hypothetical protein |
| CDR20291_3110 | 2.3412015 | conserved hypothetical protein |
| CDR20291_3387 | 2.336615 | conserved hypothetical protein |
| CDR20291_3458 | 2.3194883 | putative conjugative transposon FtsK_SpoIIIE-related protein |
| CDR20291_2106 | 2.315871 | putative oxidoreductase, ferredoxin subunit |
| *cspA* | 2.3125906 | cold shock protein |
| CDR20291_1336 | 2.3109813 | hypothetical protein |
| CDR20291_2709 | 2.3093321 | transposase |
| CDR20291_3285 | 2.3065357 | putative uncharacterized protein flags: precursor |
| CDR20291_3108 | 2.304577 | conserved hypothetical protein |
| CDR20291_1672 | 2.2980769 | putative arsenate reductase |
| CDR20291_3448 | 2.293609 | hypothetical protein |
| CDR20291_2916 | 2.2925122 | phosphosugar-binding transcriptional regulator |
| CDR20291_1191 | 2.2901857 | putative lantibiotic ABC transporter, ATP-binding protein |
| CDR20291_0418 | 2.289149 | hypothetical protein |
| CDR20291_2712 | 2.2884893 | putative peptidase |
| CDR20291_2361 | 2.2881973 | putative exported protein |
| CDR20291_0155 | 2.286317 | putative membrane-associated CAAX amino terminal protease |
| *d1* | 2.2808263 | conserved hypothetical protein |
| CDR20291_3293 | 2.2803311 | putative exported protein |
| CDR20291_1229 | 2.2741683 | GntR-family transcriptional regulator |
| CDR20291_2375 | 2.2706938 | putative lipoprotein |
| CDR20291_3462 | 2.2694504 | conjugative transposon protein |
| CDR20291_2977 | 2.2647276 | transcription antiterminator |
| CDR20291_0665 | 2.2631786 | putative exported protein |
| *oraE* | 2.2565958 | D-ornithine aminomutase E component |
| CDR20291_0511 | 2.253925 | conserved hypothetical protein |
| CDR20291_0775 | 2.2506297 | putative nuclease |
| *glvC* | 2.2502027 | PTS system, IIbc component |
| CDR20291_3400 | 2.2420852 | putative spore cortex-lytic enzyme |
| CDR20291_1928 | 2.2351036 | putative membrane protein |
| CDR20291_1176 | 2.2335684 | MarR-family transcriptional regulator |
| CDR20291_1481 | 2.2287 | conserved hypothetical protein |
| CDR20291_2499 | 2.2240226 | hypothetical protein |
| CDR20291_2651 | 2.2223415 | putative lipoprotein |
| CDR20291_3467 | 2.2171688 | conjugative transposon protein |
| *sspB* | 2.2168262 | small acid-soluble spore protein B |
| CDR20291_1142 | 2.2137163 | hypothetical protein |
| CDR20291_3120 | 2.21356 | putative phosphateABC transporter, permease protein |
| *spoIIIAA* | 2.2119963 | stage III sporulation protein AA |
| CDR20291_2808 | 2.2068152 | conserved hypothetical protein (fragment) |
| CDR20291_3504 | 2.204511 | PTS system, IIc component |
| CDR20291_0543 | 2.2003973 | conserved hypothetical protein |
| CDR20291_1461 | 2.19731 | holin |
| CDR20291_3417 | 2.193156 | conserved hypothetical protein |
| *glvR* | 2.1895297 | phosphosugar-binding transcriptional regulator |
| CDR20291_1527 | 2.1887293 | putative membrane protein |
| CDR20291_0956 | 2.1862566 | hypothetical protein |
| *srlB* | 2.179912 | PTS system, IIa component |
| CDR20291_1707 | 2.1784844 | putative two-component system response regulator |
| *fur* | 2.1756976 | ferric uptake regulation protein |
| *licT* | 2.1745243 | putative transcription antiterminator |
| CDR20291_2490 | 2.1732683 | putative response regulator |
| CDR20291_0560 | 2.1706605 | hypothetical protein |
| CDR20291_0697 | 2.1640105 | transposase-like protein b |
| CDR20291_1639 | 2.1629908 | putative ferrous iron transport protein A |
| CDR20291_2087 | 2.1621017 | putative aromatic compounds hydrolase |
| CDR20291_1559 | 2.1569982 | conserved hypothetical protein |
| CDR20291_1914 | 2.1558685 | hypothetical protein |
| *rbsA* | 2.1556127 | ribose ABC transporter, ATP-binding protein |
| CDR20291_2917 | 2.1528287 | putative membrane protein |
| CDR20291_3064 | 2.1512 | putative membrane protein |
| CDR20291_2182 | 2.1462066 | hypothetical protein |
| CDR20291_0512 | 2.144096 | hypothetical protein |
| CDR20291_3009 | 2.1413686 | hypothetical protein |
| CDR20291_1225 | 2.1394012 | putative phage regulatory protein |
| CDR20291_0859 | 2.1387832 | conserved hypothetical protein |
| CDR20291_1447 | 2.1384666 | putative uncharacterized protein |
| CDR20291_0796 | 2.1379225 | putative membrane protein |
| CDR20291_2281 | 2.1370592 | putative membrane protein precursor |
| CDR20291_0571 | 2.13593 | putative peptidase |
| CDR20291_2320 | 2.1343033 | putative oxidoreductase subunit |
| *spoIIR* | 2.1340494 | stage II sporulation protein |
| CDR20291_1323 | 2.133574 | putative ruberythrin |
| CDR20291_3106 | 2.1280892 | probable polysaccharide deacetylase |
| CDR20291_1496 | 2.1274724 | putative endonuclease |
| CDR20291_3461 | 2.1273339 | chloramphenicol o-acetyltransferase |
| CDR20291_3408 | 2.1271412 | hypothetical protein |
| CDR20291_0207 | 2.123493 | PTS system, IIa component |
| CDR20291_2979 | 2.1226456 | putative sugar-bisphosphate aldolase |
| CDR20291_2286 | 2.122154 | conserved hypothetical protein |
| CDR20291_0340 | 2.1204524 | hypothetical protein |
| CDR20291_1610 | 2.120411 | conserved hypothetical protein |
| CDR20291_2336 | 2.120107 | putative sigma 54 modulation protein |
| *veg* | 2.1170819 | conserved hypothetical protein |
| CDR20291_3154 | 2.113426 | hypothetical protein |
| CDR20291_3444 | 2.1119018 | ferredoxin |
| *adhE* | 2.1084027 | aldehyde-alcohol dehydrogenase |
| *srlA* | 2.1078258 | PTS system, glucitol/sorbitol-specific IIc2 component |
| CDR20291_2939 | 2.1050506 | PTS system, IIbc component pts system, iibc component precursor |
| CDR20291_0209 | 2.0995953 | PTS system, IIb component |
| CDR20291_0614 | 2.099349 | conserved hypothetical protein |
| CDR20291_0029 | 2.0985513 | putative transcription antiterminator |
| CDR20291_1738 | 2.0930097 | conserved hypothetical protein |
| CDR20291_2649 | 2.0913396 | putative N-acetylmuramoyl-L-alanine amidase |
| CDR20291_2192 | 2.0892425 | transposase (fragment) |
| CDR20291_0184 | 2.086086 | putative cell wall hydrolase |
| CDR20291_2256 | 2.0859647 | conserved hypothetical protein |
| CDR20291_0158 | 2.0842288 | putative two-component response regulator |
| CDR20291_3350 | 2.081579 | pilin |
| CDR20291_2952 | 2.0789118 | putative amidohydrolase |
| CDR20291_2690 | 2.0775318 | putative protein translocase subunit |
| CDR20291_2856 | 2.0749261 | conserved hypothetical protein |
| *rbsC* | 2.070177 | ribose ABC transporter, permease protein |
| CDR20291_0942 | 2.06901 | PTS system, IIb component |
| CDR20291_1813 | 2.0665524 | putative regulatory protein |
| CDR20291_2285 | 2.0649927 | putative membrane protein |
| CDR20291_2066 | 2.0603025 | putative membrane protein |
| CDR20291_2233 | 2.058511 | putative membrane protein |
| CDR20291_0344 | 2.0582306 | two-component response regulator |
| CDR20291_0760 | 2.0540824 | putative membrane protein |
| CDR20291_3214 | 2.049898 | hypothetical protein |
| CDR20291_0928 | 2.048106 | putative phosphatTse |
| CDR20291_0287 | 2.0476503 | PTS system, IIa component |
| CDR20291_2372 | 2.0469162 | conserved hypothetical protein |
| CDR20291_0022 | 2.044626 | putative beta-xylosidase |
| CDR20291_3498 | 2.0391083 | LysR-family transcriptional regulator |
| *abgT* | 2.0381641 | putative aminobenzoyl-glutamate transport protein |
| CDR20291_2498 | 2.0380943 | TetR-family trancscriptional regulator |
| *eutN* | 2.0369942 | putative ethanolamine/propanediol utilization protein |
| CDR20291_0171 | 2.0355306 | putative redox-sensing transcriptional repressor |
| CDR20291_1258 | 2.033891 | conserved hypothetical protein |
| CDR20291_1025 | 2.033765 | conserved hypothetical protein |
| CDR20291_3356 | 2.0323858 | putative exported protein |
| CDR20291_0514 | 2.0303247 | transposase-like protein b pseudogene |
| CDR20291_2868 | 2.0281112 | thioredoxin |
| CDR20291_0875 | 2.0269256 | conserved hypothetical protein |
| CDR20291_1619 | 2.0243516 | putative transcriptional regulator |
| CDR20291_0021 | 2.0238392 | putative beta-glucosidase |
| CDR20291_1138 | 2.021514 | conserved hypothetical protein |
| *sleB* | 2.0209548 | putative spore-cortex-lytic protein |
| CDR20291_0133 | 2.0206575 | putative transcription antiterminator |
| *cspD* | 2.0195987 | cold shock protein |
| *rpoD2* | 2.0195546 | RNA polymerase sigma factor rpoD |
| CDR20291_1472 | 2.0193841 | putative exported protein |
| CDR20291_3480 | 2.0188391 | conserved hypothetical protein |
| *spoIIIAC* | 2.0182188 | stage III sporulation protein AC |
| *spoVT* | 2.015846 | stage V sporulation protein T |
| CDR20291_0385 | 2.0120325 | putative oxidoreductase |
| CDR20291_2798 | 2.0021243 | conserved hypothetical protein |
| CDR20291_2545 | 2.0008383 | putative membrane protein |
| CDR20291_1241 | 2.0001361 | putative membrane protein |
