# Supplementary material for: Heme sensing and detoxification by HatRT contributes to pathogenesis during Clostridium difficile infection
Source: PLoS Pathog. 2018 Dec 21;14(12):e1007486. doi: 10.1371/journal.ppat.1007486 (PMC6303022; doi:10.1371/journal.ppat.1007486)
Supplement: S4 Table — (DOCX) [file ppat.1007486.s008.docx]

**S4 Table *C. difficile* R20291 genes transcriptionally downregulated in the presence of heme.**

| **Gene Symbol** | **Fold-change Heme Treated vs Untreated Media Control** | **Description** |
| --- | --- | --- |
| *cotJB1* | -8.493219 | putative spore-coat protein |
| CDR20291_0521 | -5.746132 | hypothetical protein |
| *cotJC1* | -3.9827776 | putative spore-coat protein |
| *rpsE* | -3.8330042 | 30S ribosomal protein S5 |
| *rplV* | -3.7833204 | 50S ribosomal protein L22 |
| CDR20291_3239 | -3.7300813 | ABC transporter, ATP-binding protein |
| CDR20291_3046 | -3.705808 | MerR-family transcriptional regulator |
| *rplP* | -3.7040238 | 50S ribosomal protein L16 |
| *rpsH* | -3.6383638 | 30S ribosomal protein S8 |
| *rpsS* | -3.6113067 | 30S ribosomal protein S19 |
| *rplR* | -3.604462 | 50S ribosomal protein L18 |
| *rpsQ* | -3.5911934 | 30S ribosomal protein S17 |
| *rpsN* | -3.5626237 | 30S ribosomal protein S14 |
| *rplN* | -3.5618937 | 50S ribosomal protein L14 |
| *cotJB2* | -3.5552454 | putative spore-coat protein |
| *feoB1* | -3.4932115 | ferrous iron transport protein B |
| CDR20291_1075 | -3.434818 | putative exported protein |
| *rplX* | -3.4215739 | 50S ribosomal protein L24 |
| *rpsC* | -3.3353822 | 30S ribosomal protein S3 |
| *rplE* | -3.283783 | 50S ribosomal protein L5 |
| *rplD* | -3.1962414 | 50S ribosomal protein L4 |
| *rpmD* | -3.1648352 | 50S ribosomal protein L30 |
| *rplW* | -3.136498 | 50S ribosomal protein L23 |
| *rplF* | -3.1316626 | 50S ribosomal protein L6 |
| *rpmC* | -3.102201 | 50S ribosomal protein L29 |
| *rplB* | -3.0742095 | 50S ribosomal protein L2 |
| CDR20291_3237 | -3.0330143 | ABC transporter, permease protein |
| *rplC* | -3.0299876 | 50S ribosomal protein L3 |
| CDR20291_1648 | -2.9830742 | putative ABC transporter, permease protein |
| *fhuG* | -2.8928006 | putative ferrichrome ABC transporter, permease protein |
| CDR20291_0926 | -2.8338807 | hypothetical protein |
| *adk* | -2.8257287 | adenylate kinase |
| *rpsK* | -2.818008 | 30S ribosomal protein S11 |
| CDR20291_1150 | -2.7863884 | putative ribosomal protein |
| CDR20291_1210 | -2.765053 | putative phage protein |
| CDR20291_1149 | -2.7456932 | conserved hypothetical protein |
| *ribA* | -2.7125893 | riboflavin biosynthesis protein |
| CDR20291_3099 | -2.6862726 | conserved hypothetical protein |
| *infB* | -2.6825497 | translation initiation factor IF-2 |
| *sodA* | -2.6655688 | putative superoxide dismutase [Mn] |
| CDR20291_0922 | -2.635544 | hypothetical protein |
| *tuaG* | -2.6347167 | putative teichuronic acid biosynthesis glycosyl transferase |
| CDR20291_0516 | -2.6060565 | putative cation transporting ATPase |
| CDR20291_3238 | -2.6003761 | ABC transporter, permease protein |
| CDR20291_1212 | -2.585924 | putative phage cell wall hydrolase |
| CDR20291_2449 | -2.5827234 | conserved hypothetical protein |
| CDR20291_0996 | -2.5810926 | conserved hypothetical protein |
| *fhuC* | -2.5564084 | putative ferrichrome ABC transporter, ATP-binding protein |
| *dapA2* | -2.5528688 | dihydrodipicolinate synthase |
| *fhuB* | -2.5447285 | putative ferrichrome ABC transporter, permease protein |
| CDR20291_1647 | -2.542131 | ABC transporter, ATP-binding protein |
| *sigH* | -2.5233936 | RNA polymerase sigma-H factor |
| CDR20291_3100 | -2.5013387 | conserved hypothetical protein |
| CDR20291_2138 | -2.492333 | putative sodium:solute symporter |
| *flgG* | -2.4588897 | flagellar basal-body rod protein FlgG |
| CDR20291_2289 | -2.437157 | hypothetical protein |
| *aroC* | -2.420682 | chorismate synthase (5-enolpyruvylshikimate-3-phosphate phospholyase) |
| *rpsM* | -2.4126081 | 30S ribosomal protein S13 |
| CDR20291_3022 | -2.409462 | conserved hypothetical protein |
| CDR20291_1329 | -2.384787 | putative exported protein |
| CDR20291_1538 | -2.3766038 | conserved hypothetical protein |
| CDR20291_1581 | -2.371138 | putative membrane protein |
| *thiK* | -2.3697405 | 4-methyl-5-beta-hydroxyethylthiazole kinase hydroxyethylthiazole kinase |
| CDR20291_1214 | -2.3692572 | phage protein |
| CDR20291_1649 | -2.3312683 | putative ABC transporter, permease protein |
| CDR20291_1216 | -2.329888 | phage protein |
| CDR20291_2661 | -2.3286161 | putative beta-glycosyltransferase |
| CDR20291_1211 | -2.3246927 | putative phage protein |
| CDR20291_0346 | -2.3085475 | conserved hypothetical protein |
| *metN* | -2.2932603 | D-methionine ABC transporter, ATP-binding protein |
| CDR20291_2770 | -2.2888985 | putative drug/sodium antiporter |
| *rpsD* | -2.2606547 | 30S ribosomal protein S4 |
| *flgE* | -2.2575037 | flagellar hook protein |
| *fchA* | -2.246807 | methenyltetrahydrofolate cyclohydrolase |
| *obg* | -2.246415 | Spo0B-associated GTP-binding protein |
| *csrA* | -2.2385561 | carbon storage regulator |
| *rpmJ* | -2.233 | 50S ribosomal protein L36 |
| CDR20291_3006 | -2.2250257 | putative phage protein |
| *dapB1* | -2.2225766 | dihydrodipicolinate reductase |
| CDR20291_0746 | -2.2034466 | probable transporter |
| CDR20291_0043 | -2.197775 | thymidylate synthase |
| *fabG* | -2.1970513 | 3-oxoacyl-[acyl-carrier protein] reductase |
| *gutA* | -2.196997 | PTS system, glucitol/sorbitol-specific IIc2 component |
| CDR20291_0273 | -2.1895223 | putative flagellar basal-body rod protein |
| *flgL* | -2.1887264 | flagellar hook-associated protein |
| *acoB* | -2.1874819 | acetoin:2,6-dichlorophenolindophenol oxidoreductase beta subunit |
| CDR20291_0228 | -2.1758933 | conserved hypothetical protein |
| *asd* | -2.1721008 | aspartate-semialdehyde dehydrogenase |
| CDR20291_2814 | -2.1702242 | conserved hypothetical protein |
| *rpsJ* | -2.16123 | 30S ribosomal protein S10 |
| *hisS* | -2.1584945 | putative histidyl-tRNA synthetase |
| CDR20291_0045 | -2.146511 | putative uncharacterized protein |
| CDR20291_0047 | -2.1418478 | putative thymidylate synthase |
| CDR20291_2662 | -2.1404283 | putative teichuronic acid biosynthesis glycosyl transferase |
| CDR20291_1217 | -2.1344755 | putative phage tail fiber protein |
| *uvrB* | -2.1312566 | excinuclease ABC subunit B |
| *iunH* | -2.1290586 | inosine-uridine preferring nucleoside hydrolase |
| CDR20291_1551 | -2.1245148 | putative lipoprotein |
| *rplO* | -2.1228526 | 50S ribosomal protein L15 |
| CDR20291_1125 | -2.1123612 | putative holliday junction resolvase |
| CDR20291_0804 | -2.1063793 | ABC transporter, permease protein |
| *feoA1* | -2.1019013 | putative ferrous iron transport protein A |
| *cheC* | -2.0987973 | chemotaxis protein CheC |
| *sip2* | -2.0981913 | singal peptidase I |
| *nusA* | -2.0957475 | transcription elongation protein |
| CDR20291_0995 | -2.0921319 | radical SAM-superfamily protein |
| *hadB* | -2.0901814 | subunit of oxygen-sensitive 2-hydroxyisocaproyl-CoA dehydratase |
| *tpi* | -2.0859563 | triosephosphate isomerase |
| CDR20291_0648 | -2.0853581 | conserved hypothetical protein |
| CDR20291_1218 | -2.085296 | putative phage protein |
| CDR20291_3102 | -2.0845835 | hypothetical protein |
| *rnfE* | -2.0844994 | electron transport complex protein |
| *rnfA* | -2.0843024 | electron transport complex protein |
| *fleN* | -2.0812418 | flagellar number regulator |
| CDR20291_2884 | -2.0809321 | putative PTS system, IIb component |
| CDR20291_2346 | -2.0776253 | conserved hypothetical protein |
| *folD* | -2.0760767 | putative FolD bifunctional protein |
| *prlA* | -2.0753083 | preprotein translocase SecY subunit |
| CDR20291_1213 | -2.0732968 | hypothetical protein |
| CDR20291_0065 | -2.0723321 | elongation factor TU |
| CDR20291_0985 | -2.0693574 | putative penicillin-binding protein |
| CDR20291_0044 | -2.061752 | dihydrofolate reductase region |
| *fliS1* | -2.0603256 | flagellar protein FliS |
| CDR20291_2349 | -2.0602372 | ABC transporter, ATP-binding protein |
| *manC* | -2.0563254 | putative mannose-1-phosphate guanylyltransferase |
| CDR20291_2526 | -2.0555916 | two-component response regulator |
| CDR20291_0049 | -2.0538368 | conserved hypothetical protein |
| *pgm2* | -2.0533345 | putative phosphomannomutase/phosphoglycerate mutase |
| CDR20291_1103 | -2.0531716 | putative FMN-dependent dehydrogenase |
| *map1* | -2.0526085 | methionine aminopeptidase |
| CDR20291_2825 | -2.0481207 | ABC transporter, ATP-binding protein |
| CDR20291_0227 | -2.0478148 | putative transglycosylase |
| *rpiB1* | -2.0455568 | ribose-5-phosphate isomerase 1 |
| *cheD* | -2.0426855 | chemotaxis protein |
| *hadI* | -2.034394 | activator of 2-hydroxyisocaproyl-CoA dehydratase |
| *mreC* | -2.029631 | putative rod shape-determining protein precursor |
| CDR20291_1615 | -2.0271087 | probable permease |
| *pheT* | -2.023367 | phenylalanyl-tRNA synthetase beta chain |
| CDR20291_1107 | -2.0229175 | putative ABC transporter, permease protein |
| *thiI* | -2.0227945 | putative thiamine biosynthesis protein |
| CDR20291_2911 | -2.0177546 | restriction modification system dna specificity domain |
| CDR20291_0463 | -2.0165677 | putative methyl accpeting chemotaxis protein |
| *feoB3* | -2.0153618 | putative ferrous iron transport protein B |
| *aspS* | -2.010659 | putative aspartyl-tRNA synthetase |
| *rpe* | -2.0086982 | putative ribulose-phosphate 3-epimerase |
| *flgD* | -2.00112 | putative basal-body rod modification protein |
